# MicroRNA-374a, -4680, and -133b suppress cell proliferation through the regulation of genes associated with human cleft palate in cultured human palate cells

**DOI:** 10.1186/s12920-019-0546-z

**Published:** 2019-07-01

**Authors:** Akiko Suzuki, Aimin Li, Mona Gajera, Nada Abdallah, Musi Zhang, Zhongming Zhao, Junichi Iwata

**Affiliations:** 1 Department of Diagnostic & Biomedical Sciences, School of Dentistry, The University of Texas Health Science Center at Houston, Houston, TX USA; 2 Center for Craniofacial Research, The University of Texas Health Science Center at Houston, Houston, TX USA; 3 Center for Precision Health, School of Biomedical Informatics, The University of Texas Health Science Center at Houston, Houston, TX USA; 4 School of Computer Science and Engineering, Xi’an University of Technology, Xi’an, 710048 Shaanxi China; 5 MD Anderson Cancer Center UTHealth Graduate School of Biomedical Sciences, 1941 East Road, BBS 4208, Houston, TX 77054 USA

**Keywords:** Cleft palate, Bioinformatics, Gene mutation, microRNA, KEGG pathway, Gene ontology

## Abstract

**Background:**

Cleft palate (CP) is the second most common congenital birth defect; however, the relationship between CP-associated genes and epigenetic regulation remains largely unknown. In this study, we investigated the contribution of microRNAs (miRNAs) to cell proliferation and regulation of genes involved in CP development.

**Methods:**

In order to identify all genes for which mutations or association/linkage have been found in individuals with CP, we conducted a systematic literature search, followed by bioinformatics analyses for these genes. We validated the bioinformatics results experimentally by conducting cell proliferation assays and miRNA-gene regulatory analyses in cultured human palatal mesenchymal cells treated with each miRNA mimic.

**Results:**

We identified 131 CP-associated genes in the systematic review. The bioinformatics analysis indicated that the CP genes were associated with signaling pathways, microRNAs (miRNAs), metabolic pathways, and cell proliferation. A total 17 miRNAs were recognized as potential modifiers of human CP genes. To validate miRNA function in cell proliferation, a main cause of CP, we conducted cell proliferation/viability assays for the top 11 candidate miRNAs from our bioinformatics analysis. Overexpression of miR-133b, miR-374a-5p, and miR-4680-3p resulted in a more than 30% reduction in cell proliferation activity in human palatal mesenchymal cell cultures. We found that several downstream target CP genes predicted by the bioinformatics analyses were significantly downregulated through induction of these miRNAs (*FGFR1*, *GCH1*, *PAX7*, *SMC2*, and *SUMO1* by miR-133b; *ARNT*, *BMP2*, *CRISPLD1*, *FGFR2*, *JARID2*, *MSX1*, *NOG*, *RHPN2*, *RUNX2*, *WNT5A* and *ZNF236* by miR*-*374a-5p; and *ERBB2*, *JADE1*, *MTHFD1* and *WNT5A* by miR-4680-3p) in cultured cells.

**Conclusions:**

Our results indicate that miR-374a-5p, miR-4680-3p, and miR-133b regulate expression of genes that are involved in the etiology of human CP, providing insight into the association between CP-associated genes and potential targets of miRNAs in palate development.

**Electronic supplementary material:**

The online version of this article (10.1186/s12920-019-0546-z) contains supplementary material, which is available to authorized users.

## Background

Cleft lip with/without cleft palate (CL/CP) is the second most common birth defect in humans worldwide [1]. CP includes both cleft lip with cleft palate (CLP) and isolated cleft palate (*aka* cleft palate only, CPO). Prevalence of CP is estimated to be approximately 1/500 to 1/2500 live births, with ethnic and geographic variations (the highest prevalence is seen in Asian and Native American populations, and the lowest in African-derived populations) [1–3]. Approximately 70% of CLP and 50% of CPO cases are non-syndromic (i.e. there is no deformity in other parts of the body), and the remainder are syndromic (CP is part of the clinical features of the condition) [4–7]. Previous studies have identified a large number of gene mutations, chromosomal abnormalities, and teratogens in CP [1, 2]. In addition to genetic mutations, genetic background (e.g. ethnicity, population of origin, and gender), substantially influences CP prevalence. Maternal age, smoking, alcohol consumption, obesity, and micronutrient deficiencies are known, or strongly suspected, experimental risk factors for CP. Therefore, the etiology of CP is complex, and its risk factors are still being elucidated [8–10]. Recent studies suggest that environmental factors control gene expression at the post-transcriptional level through epigenetic factors [11], including microRNAs (miRNAs), which are short noncoding RNAs [12].

In this study, we identified the networks and pathways of CP-associated genes and miRNAs potentially involved in the pathology of human CP, through bioinformatics analyses of CP-associated genes and subsequent experimental validation of miRNAs that regulate cell proliferation and expression of CP-associated genes in cultured human palatal mesenchymal cells.

## Methods

### Eligibility criteria for the systematic review

This systematic review followed the PRISMA (Preferred Reporting Items for Systematic reviews and Meta-Analyses) guideline and corresponding checklist. The criteria for including publications were the following: 1) articles described genes associated with human CP; 2) were published as original articles; and 3) were published in English. The exclusion criteria were the following: 1) gene mutations were not described; 2) CP was not involved; 3) CP was caused by environmental factors.

### Information sources and search

The Medline (Ovid), PubMed (National Library of Medicine), and EMBASE (Ovid) databases were used for the online searches. Any exceptional studies missed by the database searches were retrieved by a Scopus (Elsevier) search. The bibliographies of highly pertinent articles were examined to avoid any errors in the systematic review. RefWorks (Proquest) and Primary Excel Workbook were used to track all the search strategies and results for the screening of the titles and abstracts of papers found in the database search, as previously described [13]. All data and codebooks related to the systematic review were documented in the Primary Excel Workbook.

### Category enrichment analysis

Category enrichment analysis was performed using the Kyoto Encyclopedia of Genes and Genomes (KEGG) database and the WebGestalt tool, as previously described [14]. Gene sets with a false discovery rate-adjusted *p*-value < 0.05 and at least four human CP genes were considered as significantly enriched categories. The Gene Ontology (GO) database [15] was used to identify categories enriched with a significant number of human CP genes, as previously described [14].

### miRNA-target gene analysis

The miRTarbase, a database for experimentally validated miRNA-gene interactions, and three databases (miRanda, PITA, and TargetScan) for predicted miRNA-gene interactions were used to verify the miRNA-gene relationships, as previously described [14].

### Cell culture

Human palatal mesenchymal cells (HEPM cells, American Type Culture Collection) were cultured in Minimum Essential Medium Eagle-alpha modification (αMEM) supplemented with 10% fetal bovine serum (FBS), penicillin/streptomycin, and L-glutamine. The cells were plated onto 96-well cell culture plates at a density of 10,000/well and treated with a mimic for negative control, miR-27a-3p, miR-27b-3p, miR-133b, miR-203a-3p, miR-300-3p, miR-374a-5p, miR-374b-5p, miR-381-3p, miR-495-3p, miR-4680-3p, and miR-7854-3p (mirVana miRNA mimic, ThermoFisher Scientific) using the TransIT-X2 system (Mirus Bio LLC, Madison, WI), according to the manufacturer’s protocol. Cell proliferation assays were conducted using the cell counting kit 8 (Dojindo Molecular Technologies, Gaithersburg, MD) (*n* = 6 per group).

### Quantitative RT-PCR

Total RNA was extracted from HEPM cells (n = 6 per group) with the QIAshredder and RNeasy mini extraction kit (QIAGEN) or the miRNeasy mini extraction kit (QIAGEN), as previously described [16]. The sequences of the PCR primers are shown in Additional file 1: Table S1.

### Statistical analysis

A *p* value < 0.05 in two-tailed student’s *t* tests was considered to be statistically significant. All the data were parametric and were represented as mean ± standard deviation, as previously described [16].

## Results

### Literature search

**Fig. 1:**
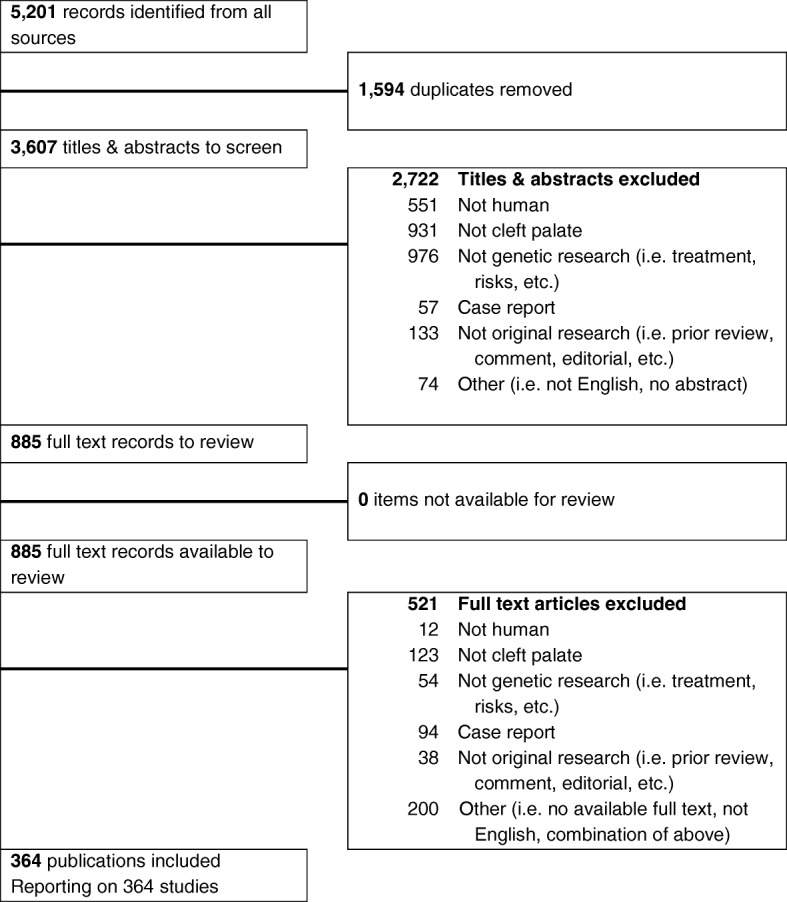
PRISMA flowchart for study selection. A graphical representation of the flow of citations reviewed in the course of the systematic review was generated using a PRISMA flow diagram

A total of 5201 articles were identified in the systematic review, and 1594 duplicates were removed. The remaining 3607 articles were screened, using the titles and abstracts, independently by two screeners; 2722 papers were excluded based on the exclusion criteria. A total of 885 papers were further assessed through full-text review: 364 studies met all inclusion criteria, and 521 articles were excluded. As a result, we identified 364 studies eligible to identify genetic mutations associated with CP (Fig. 1). After collecting data from the search engines, we performed a one-by-one literature review to obtain an accurate list of human genes involved in CLP and CPO. From these 364 studies, we identified 131 genes as human CP-associated genes (Additional file 2: Table S2, additional file 3: Table S3 and Additional file 4; Table S4).

### KEGG pathway analysis

Our central hypothesis is that genes associated with CP share common features among wide arrays of functions and pathways. To define functions, pathways, and networks crucial for palatal formation, we performed bioinformatics analyses of the genes from our gene list. The regulator pathway annotation was performed based on scoring and visualization of the pathways collected in the KEGG database. To summarize the cellular functions of genes from our list, we performed category enrichment analysis for a variety of functional relations. Among KEGG pathways, 28 pathways were significantly enriched with genes from the curated gene list (Table 1 and Additional file 5: Table S5). Eight of these pathways were related to cellular signaling: mitogen-activated protein kinase (MAPK) signaling pathway (16 genes), phosphatidylinositol 3′-kinase (PI3K)-Akt signaling pathway (16 genes), Rap1 signaling pathway (15 genes), Ras signaling pathway (15 genes), Hippo signaling pathway (15 genes), signaling pathways regulating pluripotency of stem cells (14 genes), WNT (Wingless-type MMTV integration site family) signaling pathway (7 genes), and transforming growth factor beta (TGFβ) signaling pathway (7 genes). The other two pathways were related to the structural aspects of cells and tissues: regulation of actin cytoskeleton (15 genes) and adherens junction (6 genes). In addition, the enrichment of two pathways suggested metabolic involvement: metabolic pathways (7 genes) and endocytosis (4 genes). While no specific metabolic pathways were indicated by the KEGG analysis, the KEGG metabolic pathway network showed that these seven genes play roles in cholesterol and steroid metabolic processes: *DHODH* in pyrimidine metabolism; *CYP1A1* in retinol metabolism and steroid hormone biosynthesis; *DHCR7* in cholesterol synthesis; *DHCR24* in steroid biosynthesis; *MTHFR* in folate metabolism; *PAFAH1B1* in ether lipid metabolism; and *NAT2* in caffeine metabolism. The remaining nine pathways included various aspects of cancer pathogenesis: pathways in cancer (32 genes), breast cancer (20 genes), melanoma (13 genes), basal cell carcinoma (10 genes), proteoglycans in cancer (10 genes), chemical carcinogenesis (9 genes), miRNAs in cancer (8 genes), prostate cancer (7 genes), and central carbon metabolism in cancer (4 genes). Interestingly, melanogenesis (6 genes) was also indicated as an enriched pathway, suggesting that the fate of cranial neural crest (CNC) cells, the majority of craniofacial mesenchymal cells and a source of melanocytes, was altered in CP.

**Table 1:** KEGG pathways enriched with a significant number of genes involved in CP

KEGG pathway	CP genes in pathway
Pathways in cancer	*DVL3;ERBB2;FGF1;FGF2;FGF3;FGF4;FGF7;FGF8;FGF9;FGF10;FGFR1;FGFR3;FGFR2;GSTP1;ARNT;LEF1;PDGFRA;PTCH1;RARA;BMP2;BMP4;TGFA;TGFB1;TGFB3;WNT5A;WNT11;WNT10A;AXIN2;FGF18;WNT3A;FGF19;CDH1*
Breast cancer	*DVL3;ERBB2;FGF1;FGF2;FGF3;FGF4;FGF7;FGF8;FGF9;FGF10;FGFR1;JAG2;LEF1;WNT5A;WNT11;WNT10A;AXIN2;FGF18;WNT3A;FGF19*
Melanoma	*FGF1;FGF2;FGF3;FGF4;FGF7;FGF8;FGF9;FGF10;FGFR1;PDGFRA;FGF18;FGF19;CDH1*
Hippo signaling pathway	*DVL3;FGF1;GDF6;LEF1;BMP2;BMP4;BMP7;TGFB1;TGFB3;WNT5A;WNT11;WNT10A;AXIN2;WNT3A;CDH1*
Basal cell carcinoma	*DVL3;LEF1;PTCH1;BMP2;BMP4;WNT5A;WNT11;WNT10A;AXIN2;WNT3A*
Signaling pathways regulating pluripotency of stem cells	*DVL3;FGF2;FGFR1;FGFR3;FGFR2;JARID2;PAX6;BMP2;BMP4;WNT5A;WNT11;WNT10A;AXIN2;WNT3A*
Rap1 signaling pathway	*FGF1;FGF2;FGF3;FGF4;FGF7;FGF8;FGF9;FGF10;FGFR1;FGFR3;FGFR2;PDGFRA;FGF18;FGF19;CDH1*
Regulation of actin cytoskeleton	*FGF1;FGF2;FGF3;FGF4;FGF7;FGF8;FGF9;FGF10;FGFR1;FGFR3;FGFR2;MYH9;PDGFRA;FGF18;FGF19*
MAPK signaling pathway	*FGF1;FGF2;FGF3;FGF4;FGF7;FGF8;FGF9;FGF10;FGFR1;FGFR3;FGFR2;PDGFRA;TGFB1;TGFB3;FGF18;FGF19*
Ras signaling pathway	*FGF1;FGF2;FGF3;FGF4;FGF7;FGF8;FGF9;FGF10;FGFR1;FGFR3;FGFR2;TBK1;PDGFRA;FGF18;FGF19*
Chemical carcinogenesis	*NAT2;ADH1C;CYP1A1;CYP1B1;GSTP1;GSTT1;ARNT;UGT1A7;NAT1*
One carbon pool by folate	*ALDH1L1;DHFR;MTHFD1;MTHFR;MTR*
PI3K-Akt signaling pathway	*COL2A1;FGF1;FGF2;FGF3;FGF4;FGF7;FGF8;FGF9;FGF10;FGFR1;FGFR3;FGFR2;NOS3;PDGFRA;FGF18;FGF19*
TGF-beta signaling pathway	*GDF6;BMP2;BMP4;BMP7;TGFB1;TGFB3;NOG*
Prostate cancer	*ERBB2;FGFR1;FGFR2;GSTP1;LEF1;PDGFRA;TGFA*
Cysteine and methionine metabolism	*AHCYL2;BHMT2;MTR;BHMT;CBS*
Proteoglycans in cancer	*ERBB2;FGF2;FGFR1;PTCH1;SDC2;TGFB1;WNT5A;WNT11;WNT10A;WNT3A*
Metabolism of xenobiotics by cytochrome P450	*ADH1C;CYP1A1;CYP1B1;GSTP1;GSTT1;UGT1A7*
Adherens junction	*ERBB2;FGFR1;LEF1;NECTIN1;NECTIN2;CDH1*
EGFR tyrosine kinase inhibitor resistance	*ERBB2;FGF2;FGFR3;FGFR2;PDGFRA;TGFA*
MicroRNAs in cancer	*CYP1B1;ERBB2;FGFR3;PDGFRA;ABCB1;TPM1;TP63;WNT3A*
Caffeine metabolism	*NAT2;NAT1*
Tryptophan metabolism	*TPH2;CYP1A1;CYP1B1;DDC*
Central carbon metabolism in cancer	*ERBB2;FGFR1;FGFR3;FGFR2;PDGFRA*
Melanogenesis	*DVL3;LEF1;WNT5A;WNT11;WNT10A;WNT3A*
Arginine biosynthesis	*ASL;ASS1;NOS3*
Wnt signaling pathway	*DVL3;LEF1;WNT5A;WNT11;WNT10A;AXIN2;WNT3A*
Biosynthesis of amino acids	*ASL;ASS1;MTR;PAH;CBS*

### GO functional enrichment analysis

We analyzed the CP genes from our curated list using the GO database resource to identify the enriched functional categories. The GO biological processes showed a strong association with morphogenesis: inner ear morphogenesis (10 genes), face morphogenesis (9 genes), embryonic limb morphogenesis (8 genes), branching involved in ureteric bud morphogenesis (6 genes), embryonic cranial skeleton morphogenesis (6 genes), and branching involved in salivary gland morphogenesis (5 genes). Further enriched terms emphasized development: palate development (13 genes), skeletal system development (10 genes), and pituitary gland development (6 genes) (Table 2 and Additional file 6: Table S6). We also identified regionalization (30 genes) as an enriched term, suggesting that the arrangement and patterning of cells play important roles in palate development. All genes identified in our literature search were involved in development and morphogenesis.

**Table 2:** GO biological process terms enriched with a significant number of genes involved in CP

GO biological process	CP genes in biological process category
GO:0045893 positive regulation of transcription, DNA-templated	*WNT5A, FGF7, WNT3A, GDF6, TGFB3, PAX6, FGF10, TP63, CDH1, PAX3, TGFB1, ARNT, FOXF2, BCL3, RARA, RUNX2, FGF2, BMP4, DVL3, BMP2, LEF1, TBX1, IRF6, IRF7, FOXE1, TFAP2A, ROR2, PTCH1, WNT11, BMP7*
GO:0014066 regulation of phosphatidylinositol 3-kinase signaling	*FGF19, FGFR2, FGF18, FGFR1, FGF8, FGF7, FGFR3, FGF9, ERBB2, PDGFRA, FGF10, FGF1, FGF2, FGF3, FGF4*
GO:0036092 phosphatidylinositol-3-phosphate biosynthetic process	*FGF19, FGFR2, FGF18, FGFR1, FGF8, FGF7, FGFR3, FGF9, FGF10, FGF1, FGF2, FGF3, FGF4*
GO:0046854 phosphatidylinositol phosphorylation	*FGF19, FGFR2, FGF18, FGFR1, FGF8, FGF7, FGFR3, FGF9, ERBB2, PDGFRA, FGF10, FGF1, FGF2, FGF3, FGF4*
GO:0008543 fibroblast growth factor receptor signaling pathway	*FGF19, FGFR2, FGF18, FGFR1, FGF8, FGF7, FGFR3, FGF9, FGF10, UBB, FGF1, FGF2, FGF3, FGF4*
GO:0048015 phosphatidylinositol-mediated signaling	*FGF19, FGFR2, FGF18, FGFR1, FGF8, FGF7, FGFR3, FGF9, ERBB2, PDGFRA, FGF10, FGF1, FGF2, FGF3, FGF4*
GO:0060021 palate development	*WNT5A, SUMO1, MSX1, GABRB3, WNT3A, FOXF2, TGFB3, LEF1, TFAP2A, COL2A1, WNT11, VAX1, COL11A2*
GO:0018108 peptidyl-tyrosine phosphorylation	*FGFR2, FGF18, FGFR1, FGF8, FGF7, FGFR3, FGF9, RYK, ERBB2, PDGFRA, FGF10, ROR2, FGF1, FGF2, FGF3, FGF4*
GO:0045944 positive regulation of transcription from RNA polymerase II promoter	*FGFR2, WNT5A, NOG, TBK1, WNT3A, TGFB3, PAX6, FGF10, TP63, PAX3, GREM1, TGFB1, ARNT, JADE1, PAX9, PAX7, FOXF2, BCL3, RARA, FGF1, FGF2, FGF4, BMP4, BMP2, MAFB, LEF1, GRHL3, TBX1, MSX1, IRF7, TFAP2A, UBB, BMP7*
GO:0051781 positive regulation of cell division	*FGFR2, FGF8, FGF7, FGF9, TGFB3, TGFA, FGF1, FGF2, TGFB1, FGF3, FGF4*
GO:0042475 odontogenesis of dentin-containing tooth	*BMP4, BMP2, MSX1, JAG2, TP63, LEF1, FGF10, TBX1, BMP7, RUNX2, FGF4*
GO:0050679 positive regulation of epithelial cell proliferation	*FGFR2, BMP4, NOG, FGF7, FGF9, ERBB2, TGFA, FGF10, TBX1, FGF1, TGFB1*
GO:0008284 positive regulation of cell proliferation	*FGFR2, FGF19, FGFR1, FGF18, FGF8, FGF7, FGFR3, FGF9, WNT3A, LEF1, TBX1, GREM1, NTN1, TGFB1, PDGFRA, TGFA, RARA, FGF1, FGF2, RUNX2, FGF3, FGF4*
GO:0060325 face morphogenesis	*NOG, MSX1, CRISPLD1, PAX9, CRISPLD2, TGFB3, LEF1, TBX1, TGFB1*
GO:0000165 MAPK cascade	*FGFR2, FGF19, FGFR1, FGF18, FGF8, FGFR3, FGF7, FGF9, ERBB2, FGF10, TGFB1, PDGFRA, UBB, FGF1, FGF2, FGF3, FGF4*
GO:0001837 epithelial to mesenchymal transition	*FGFR2, WNT5A, BMP2, NOG, FOXF2, LEF1, WNT11, BMP7, TGFB1*
GO:0042472 inner ear morphogenesis	*FGFR2, FGFR1, MAFB, FGF9, WNT3A, TFAP2A, ROR2, COL2A1, TBX1, NTN1*
GO:0002062 chondrocyte differentiation	*BMP4, FGFR1, BMP2, FGFR3, FGF9, COL2A1, COL11A2, RUNX2, TGFB1*
GO:0008285 negative regulation of cell proliferation	*BMP4, BMP2, CYP1B1, JARID2, TGFB3, FGF10, BRIP1, TIMP2, TGFB1, MSX1, IRF6, ROR2, TFAP2A, NOS3, RARA, AXIN2, BMP7, FGF2*
GO:0070374 positive regulation of ERK1 and ERK2 cascade	*FGF19, FGFR2, BMP4, FGF18, BMP2, FGF8, FGFR3, PDGFRA, FGF10, FGF1, FGF2, TGFB1, FGF4*
GO:0042060 wound healing	*WNT5A, NOG, ERBB2, PDGFRA, TGFB3, TGFA, FGF10, GRHL3, FGF2, TPM1*
GO:0001759 organ induction	*BMP4, FGFR1, FGF8, FGF10, FGF1, FGF2*
GO:0030326 embryonic limb morphogenesis	*FGFR1, FGF9, TP63, LEF1, PTCH1, SP8, GREM1, BMP7*
GO:0045892 negative regulation of transcription, DNA-templated	*WNT5A, BMP4, BMP2, JARID2, TBX22, LEF1, TP63, GREM1, TGFB1, SUMO1, PAX9, FOXF2, FOXE1, BCL3, TFAP2A, WNT11, RARA, BMP7, RUNX2*
GO:0090090 negative regulation of canonical Wnt signaling pathway	*WNT5A, JADE1, DVL3, BMP2, NOG, ROR2, LEF1, WNT11, UBB, GREM1, AXIN2, MLLT3*
GO:0042476 odontogenesis	*FGFR2, BMP4, WNT10A, FGF8, PAX9, TGFB3, AXIN2*
GO:0043547 positive regulation of GTPase activity	*FGFR2, FGF19, DVL3, FGFR1, FGF18, FGF8, FGFR3, FGF7, FGF9, ERBB2, FGF10, GRHL3, ARHGAP29, PDGFRA, WNT11, AXIN2, FGF1, FGF2, FGF3, FGF4*
GO:0009086 methionine biosynthetic process	*MTHFD1, BHMT2, MTR, BHMT, MTRR*
GO:0042487 regulation of odontogenesis of dentin-containing tooth	*BMP4, WNT10A, BMP2, FGF8, RUNX2*
GO:0030509 BMP signaling pathway	*BMP4, BMP2, NOG, FGF8, GDF6, ROR2, LEF1, BMP7, RUNX2*
GO:0001701 in utero embryonic development	*FGFR2, FGFR1, BMP2, NOG, MSX1, WNT3A, TGFB3, JAG2, PTCH1, NOS3, MYH9, TPM1*
GO:0043410 positive regulation of MAPK cascade	*FGFR2, FGFR1, BMP2, FGFR3, RYK, FGF9, FGF10, TBX1, TIMP2*
GO:0046655 folic acid metabolic process	*MTHFD1, MTHFR, ALDH1L1, DHFR, SLC19A1, MTRR*
GO:0042493 response to drug	*DVL3, MTHFR, FGF8, CYP1A1, ASS1, SLC6A4, TGFA, CDH1, PTCH1, ABCB1, ABCA1, TIMP2, GAD1, TGFB1*
GO:0010628 positive regulation of gene expression	*WNT10A, BMP2, NOG, FGF8, FGF9, WNT3A, ERBB2, SLC6A4, PAX6, TFAP2A, LEF1, WNT11, TGFB1*
GO:0003148 outflow tract septum morphogenesis	*FGFR2, BMP4, DVL3, FGF8, TBX1, RARA*
GO:0001501 skeletal system development	*FGFR1, BMP2, NOG, FGFR3, TCOF1, JAG2, TP63, COL2A1, COL11A2, BMP7*
GO:0001525 angiogenesis	*FGFR2, FGFR1, FGF18, CYP1B1, FGF9, TGFA, FGF10, TBX1, NOS3, STAB2, FGF1, MYH9*
GO:0060445 branching involved in salivary gland morphogenesis	*FGFR2, FGFR1, FGF8, FGF7, BMP7*
GO:0045165 cell fate commitment	*FGFR2, WNT5A, WNT10A, BMP2, FGF8, ROR2, WNT11*
GO:0002053 positive regulation of mesenchymal cell proliferation	*FGFR2, WNT5A, FGFR1, FGF9, TP63, TBX1*
GO:0031069 hair follicle morphogenesis	*FGFR2, WNT10A, FGF7, FOXE1, TP63, FGF10*
GO:0000122 negative regulation of transcription from RNA polymerase II promoter	*BMP4, FGFR2, FGFR1, NOG, BMP2, JARID2, FGF9, TBX22, PAX6, LEF1, TP63, VAX1, TGFB1, MSX1, IRF7, FOXE1, TFAP2A, PTCH1, RARA, UBB*
GO:0021983 pituitary gland development	*BMP4, NOG, MSX1, PAX6, FGF10, CDH1*
GO:0048701 embryonic cranial skeleton morphogenesis	*FGFR2, BMP4, PDGFRA, TFAP2A, TBX1, RUNX2*
GO:0001934 positive regulation of protein phosphorylation	*FGF19, BMP4, DVL3, BMP2, WNT3A, ERBB2, TBX1, AXIN2, TGFB1*
GO:0032355 response to estradiol	*ASS1, SLC6A4, FGF10, PTCH1, RARA, BMP7, TGFB1, GSTP1*
GO:0030501 positive regulation of bone mineralization	*BMP4, BMP2, TGFB3, TFAP2A, BMP7, TGFB1*
GO:0060395 SMAD protein signal transduction	*BMP4, BMP2, GDF6, TGFB3, ROR2, BMP7, TGFB1*
GO:0043066 negative regulation of apoptotic process	*BMP4, WNT5A, TP63, LEF1, GREM1, MSX1, PAX7, TGFA, BCL3, TFAP2A, RARA, WNT11, UBB, GSTP1, FGF4*
GO:0001657 ureteric bud development	*FGFR2, BMP4, FGFR1, RARA, BMP7, TGFB1*
GO:0001649 osteoblast differentiation	*BMP4, BMP2, NOG, FGF9, WNT3A, LEF1, WNT11, RUNX2*
GO:0071300 cellular response to retinoic acid	*WNT5A, WNT3A, SLC6A4, TBX1, RARA, WNT11, ABCA1*
GO:0001658 branching involved in ureteric bud morphogenesis	*BMP4, BMP2, FGF8, PTCH1, GREM1, FGF2*
GO:0000187 activation of MAPK activity	*WNT5A, BMP2, TGFB3, TGFA, FGF10, UBB, FGF1, FGF2*
GO:0048762 mesenchymal cell differentiation	*FGFR2, FGFR1, BMP2, BMP7*
GO:0030324 lung development	*FGFR2, WNT5A, FGF18, CRISPLD2, NOS3, FGF1, FGF2*
GO:0001843 neural tube closure	*MTHFD1, BMP4, NOG, GRHL3, PTCH1, RARA, TGFB1*
GO:0045666 positive regulation of neuron differentiation	*BMP4, FGFR1, BMP2, GDF6, RARA, TIMP2, BMP7*
GO:0010862 positive regulation of pathway-restricted SMAD protein phosphorylation	*BMP4, BMP2, GDF6, TGFB3, BMP7, TGFB1*

Among the GO molecular functions terms, there was an enrichment of molecular binding: heparin binding (12 genes), fibroblast growth factor receptor binding (9 genes), and frizzled binding (7 genes) (Table 3 and Additional file 6: Table S6). A total of 24 out of 104 genes (23%) were in the category of growth factor binding, growth factor receptor binding, SMAD binding, Frizzled binding, and beta-catenin binding, indicating that these molecules were directly involved in growth signaling pathway as ligands, receptors, and mediators. The remaining enriched terms in the molecular function included: chondrocyte differentiation (9 genes), osteoblast differentiation (8 genes), odontogenesis (7 genes), neural tube closure (7 genes), positive regulation of neuron differentiation (7 genes), and positive regulation of bone mineralization (6 genes). These enriched categories include downstream targets and modifiers of signaling pathways initiated by growth factors and morphogens.

**Table 3:** GO molecular function terms enriched with a significant number of genes involved in CP

GO molecular function	CP genes in molecular function category
GO:0046934 phosphatidylinositol-4,5-bisphosphate 3-kinase activity	*FGF19, FGFR2, FGF18, FGFR1, FGF8, FGF7, FGFR3, FGF9, ERBB2, PDGFRA, FGF10, FGF1, FGF2, FGF3, FGF4*
GO:0016303 1-phosphatidylinositol-3-kinase activity	*FGF19, FGFR2, FGF18, FGFR1, FGF8, FGF7, FGFR3, FGF9, FGF10, FGF1, FGF2, FGF3, FGF4*
GO:0008083 growth factor activity	*BMP4, FGF19, FGF18, BMP2, FGF8, FGF7, FGF9, GDF6, JAG2, TGFB3, FGF10, TGFB1, TGFA, FGF1, BMP7, FGF2, FGF3, FGF4*
GO:0005088 Ras guanyl-nucleotide exchange factor activity	*FGF19, FGFR2, FGF18, FGFR1, FGF8, FGF7, FGFR3, FGF9, ERBB2, PDGFRA, FGF10, FGF1, FGF2, FGF3, FGF4*
GO:0005104 fibroblast growth factor receptor binding	*FGF19, FGF8, FGF7, FGF9, FGF10, FGF1, FGF2, FGF3, FGF4*
GO:0004713 protein tyrosine kinase activity	*FGFR2, FGF18, FGFR1, FGF8, FGF7, FGFR3, FGF9, RYK, ERBB2, FGF10, FGF1, FGF2, FGF3, FGF4*
GO:0008201 heparin binding	*FGFR2, BMP4, FGFR1, FGF7, CRISPLD2, FGF9, FGF10, PTCH1, FGF1, BMP7, FGF2, FGF4*
GO:0005109 frizzled binding	*WNT5A, DVL3, WNT10A, RYK, WNT3A, ROR2, WNT11*
GO:0042803 protein homodimerization activity	*FGFR2, FGFR1, NOG, GDF6, SLC6A4, NECTIN1, NECTIN2, TBX1, MYH9, MID1, TGFB1, GCH1, UGT1A7, STOM, PDGFRA, TFAP2A, PCYT1A, CBS*

Among the GO cellular components terms, several terms were enriched in the lipid bilayer components of cellular membranes and correlated with the enrichment of cholesterol and sterol metabolism as shown in the KEGG pathway analysis: extracellular region (36 genes), extracellular space (27 genes), cell surface (16 genes), and proteinaceous extracellular matrix (12 genes) (Table 4 and Additional file 6: Table S6). Owing to the large number of transcription factors in our list of CP genes, transcription factor complex (10 genes) was also an enriched term. Interestingly, additional enriched terms were specific to the neuron: synapse (9 genes) and axon (6 genes). This suggests that the fate of CNC cells, a source of the central and peripheral nervous system, might be altered and that defects in nerve formation and function may cause CP in humans.

**Table 4:** GO cellular component terms enriched with a significant number of genes involved in CP

GO cellular component	CP genes in cellular component category
GO:0005576 extracellular region	*FGF19, FGFR2, WNT5A, FGF18, FGFR1, FGF8, NOG, FGFR3, FGF7, FGF9, WNT3A, GDF6, TGFB3, FGF10, COL2A1, CDH1, MMP3, TIMP2, TGFB1, CRISPLD1, CRISPLD2, COL11A2, FGF1, PRSS35, FGF2, FGF3, FGF4, BMP4, WNT10A, BMP2, NECTIN1, TCN2, NTN1, WNT11, WDR1, BMP7*
GO:0005615 extracellular space	*WNT5A, FGF18, FGF8, NOG, FGF9, GDF6, WNT3A, TGFB3, FGF10, COL2A1, GREM1, TIMP2, MMP3, TGFB1, SERPINA6, TGFA, FGF1, FGF2, BMP4, WNT10A, BMP2, TCN2, STOM, WNT11, UBB, BMP7, GSTP1*
GO:0005578 proteinaceous extracellular matrix	*WNT5A, BMP4, WNT10A, CRISPLD2, WNT3A, WNT11, COL11A2, FGF1, TIMP2, MMP3, TGFB1, MMP25*
GO:0009986 cell surface	*WNT5A, FGFR2, BMP2, FGFR3, WNT3A, TGFB3, FGF10, NECTIN2, ABCB1, GREM1, TIMP2, TGFB1, SDC2, TNS1, TGFA, RARA*

### Environmental and epigenetic factors

In addition to gene mutations, both genetic background and environmental factors influence CP prevalence. Recent studies suggest that environmental factors can regulate miRNAs that control gene expression at post-transcriptional levels [17]. To investigate how miRNAs regulate CP genes, we conducted an enrichment analysis of known miRNAs and their targets (Table 5 and Additional file 7: Table S7). With *p*-value < 0.005, our list of CP genes was significantly enriched with the targets of 18 miRNAs: hsa-miR-27a (mir-27 family; 11 CP genes), hsa-miR-27b (mir-27 family; 11 CP genes), hsa-miR-103 (mir-103 family; 8 CP genes), hsa-miR-133a (mir-133 family; 6 CP genes), hsa-miR-133b (mir-133 family; 11 CP genes), hsa-miR-148a-5p (mir-148 family; 4 CP genes), hsa-miR-203a-3p (mir-203 family; 9 CP genes), hsa-miR-300 (mir-154 family; 15 CP genes), hsa-miR-324-5p (mir-324 family; 9 CP genes), hsa-miR-374a (mir-374 family; 15 CP genes), hsa-miR-374b (mir-374 family; 15 CP genes), hsa-miR-381 (mir-154 family; 13 CP genes), hsa-miR-495 (mir-329 family; 15 CP genes), hsa-miR-3976 (unknown family; 4 CP genes), hsa-miR-4453 (unknown family; 4 CP genes), hsa-miR-4538 (unknown family; 4 CP genes), hsa-miR-4680-3p (mir-4680 family; 5 CP genes), and hsa-miR-7854-3p (unknown family; 6 CP genes). Thus, miRNAs may regulate the expression of multiple CP-associated genes and play an important role in the pathology of CP.

**Table 5:** miRNA families that target a motif in a significant number of genes involved in CP

miRNA	CP genes with target MOTIF
hsa-miR-300	*ABCA1;CRISPLD1;FGF7;FGFR2;FOXF2;GABRB3;GAD1;JAG2;LEF1;MID1;MLLT3;PTCH1;WNT5A;CRISPLD2;GREM1*
hsa-miR-381	*ABCA1;CRISPLD1;FGF7;FGFR2;FOXF2;GABRB3;GAD1;JAG2;LEF1;MID1;MLLT3;PTCH1;WNT5A*
hsa-miR-495	*ARNT;BMP2;CYP1B1;FGF1;FGF19;FGF7;GAD1;JAG2;MLLT3;NTN1;PRSS35;PTCH1;RUNX2;SUMO1;VAX1*
hsa-miR-374a	*ARNT;BMP2;CRISPLD1;FGFR2;JARID2;MSX1;NOG;NTN1;PAX6;RHPN2;RUNX2;TGFA;TNS1;WNT5A;ZNF236*
hsa-miR-374b	*ARNT;BMP2;CRISPLD1;FGFR2;JARID2;MSX1;NOG;NTN1;PAX6;RHPN2;RUNX2;TGFA;TNS1;WNT5A;ZNF236*
hsa-miR-4680-3p	*ERBB2;JADE1;MTHFD1;TBK1;WNT5A*
hsa-miR-203a-3p	*CDH1;FGF2;GREM1;PAX6;RUNX2;STOM;SUMO1;TBK1;TP63*
hsa-miR-7854-3p	*BRIP1;CBS;CRISPLD2;FGF19;FGFR1;MSX1*
hsa-miR-133b	*FGF1;FGFR1;GCH1;MLLT3;MYH9;PAX7;SMC2;STOM;SUMO1;ZNF236;GSTP1*
hsa-miR-27a	*ABCA1;BCL3;GABRB3;GCH1;GDF6;GREM1;MN1;PAX9;PDGFRA;RARA;SUMO1*
hsa-miR-27b	*ABCA1;BCL3;GABRB3;GCH1;GDF6;GREM1;MN1;PAX9;PDGFRA;RARA;SUMO1*
hsa-miR-4453	*CBS;MYH9;RYK;SP8*
hsa-miR-4538	*CBS;MYH9;RYK;SP8*
hsa-miR-103	*AXIN2;FGF2;FGF7;FGFR2;GAD1;MYH9;TPM1;WNT3A*
hsa-miR-133a	*FGF1;GCH1;MLLT3;MYH9;SUMO1;ZNF236*
hsa-miR-148a-5p	*ABCA1;CRISPLD2;CYP1B1;TNS1*
hsa-miR-324-5p	*GDF6;RUNX2;SLC6A4;ARNT;ASS1;CBS;MTHFD1;PAX3;TCOF1*
hsa-miR-3976	*AHCYL2;CYP1B1;GDF6;WDR1*

### Experimental validation

**Fig. 2:**
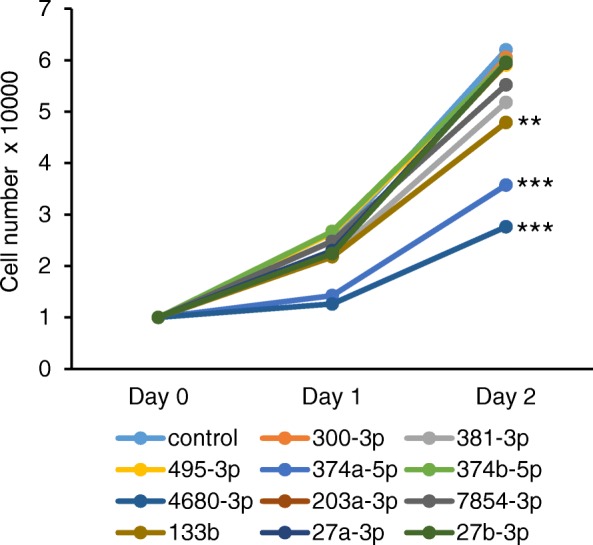
Effect of predicted miRNAs on cell proliferation. Cell proliferation assays in human palatal fibroblasts treated with the indicated miRNA mimics. Negative control (control, light blue), miR-300-3p (orange), miR-381-3p (light gray), miR-495-3p (yellow), miR-374a-5p (blue), miR-374b-5p (light green), miR-4680-3p (dark blue), miR-203a-3p (brown), miR-7854-3p (gray), miR-133b (light brown), miR-27a-3p (navy), and miR27b-3p (green). ** *p* < 0.01, *** *p* < 0.001. Each treatment group was compared with the control. *n* = 6 per group

The expression of target mRNAs is anti-correlated with miRNA expression [18]. To test whether the induction of these miRNAs caused proliferation defects through the inhibition of target genes, human palatal mesenchymal cells were treated with each miRNA mimic. The mimics for either miR-133b, miR-374a-5p or miR-4680-3p significantly inhibited (reduction of more than 30% of cell number) cell proliferation in human palatal mesenchymal cells; by contrast, treatment with mimics for miR-27a-3p, miR-27b-3p, miR-203a-3p, miR-300-3p, miR-374b-5p, and miR-495-3p resulted in no proliferation defects (Fig. 2 and Additional file 8: Table S8). The mimics for either miR-381-3p or miR-7854-3p slightly inhibited (an approximate reduction of 10%) cell proliferation.

**Fig. 3:**
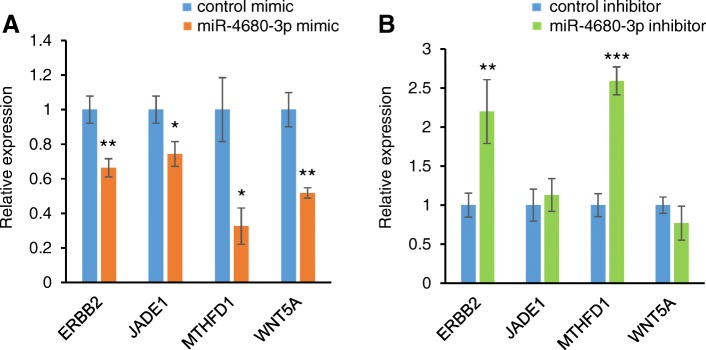
Effect of miR-4680-3p on predicted target genes. **a** Quantitative RT-PCR for the indicated genes after treatment with negative control (light blue) or miR-4680-3p mimic (orange). * *p* < 0.05, ** *p* < 0.01. Each treatment group was compared with the control. n = 6 per group. **b** Quantitative RT-PCR for the indicated genes after treatment with negative control (light blue) or miR-4680-3p inhibitor (light green). ** *p* < 0.01, *** *p* < 0.001. Each treatment group was compared with the control. n = 6 per group

To identify target genes regulated by miR-133b, miR-374a-5p, and miR-4680-3p, we conducted quantitative RT-PCR analyses for the predicted target genes (*FGF1*, *FGFR1*, *GCH1*, *GSTP1, MLLT3*, *MYH9*, *PAX7*, *SMC2*, *STOM*, *SUMO1*, and *ZNF236* for hsa-miR-133b; *ARNT*, *BMP2*, *CRISPLD1*, *FGFR1*, *JARID2*, *MSX1*, *NOG*, *NTN1*, *RHPN2*, *RUNX2*, *TNS1*, *WNT5A*, and *ZNF236* for hsa-miR-374a-5p; and *ERBB2*, *JADE1*, *MTHFD1*, and *WNT5A* for hsa-miR-4680-3p) in human palatal mesenchymal cells treated with either miR-133b, miR-374a-5p, or miR-4680-3p. *PAX6* and *TGFA* were excluded from the gene expression experiments because *Pax6* is expressed only in the cephalic ectoderm [19] and *TGFA* is expressed at the medial edge epithelium of the fusing palatal shelves [20, 21]. The expression of *ERBB2*, *JADE1*, *MTHFD1* and *WNT5A* was significantly downregulated in cultured human palatal mesenchymal cells treated with miR-4680-3p mimic (Fig. 3a). To further evaluate the anti-correlation of miRNAs and target genes, we treated cells with a miR-4680-3p inhibitor and found that expression of *ERBB2* and *MTHFD1* was significantly upregulated (Fig. 3b). Therefore, these results indicate that *ERBB2* and *MTHFD1* are downstream target genes of miR-4680-3p in cultured human palate cells.

**Fig. 4:**
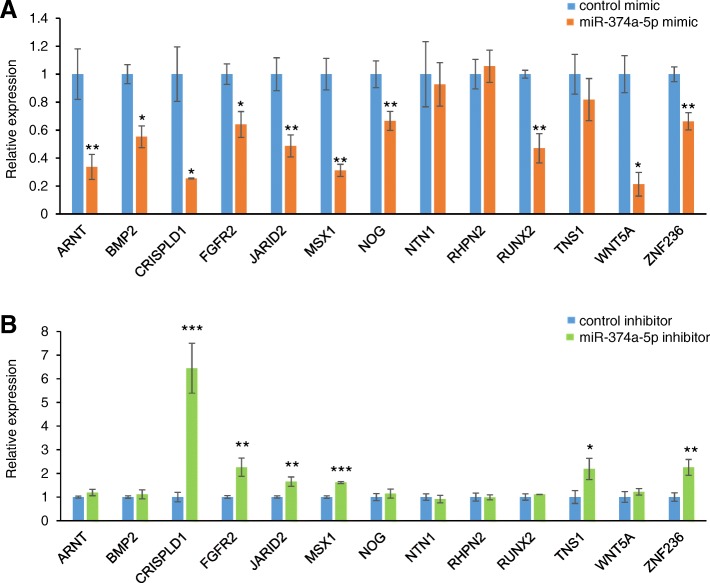
Effect of miR-374a-5p on predicted target genes. **a** Quantitative RT-PCR for the indicated genes after treatment with negative control (light blue) or miR-374a-5p mimic (orange). * *p* < 0.05, ** *p* < 0.01, *** *p* < 0.001. Each treatment group was compared with the control. n = 6 per group. **b** Quantitative RT-PCR for the indicated genes after treatment with negative control (light blue) or miR-374a-5p inhibitor (light green). * *p* < 0.05, ** *p* < 0.01, *** *p* < 0.001. Each treatment group was compared with the control. n = 6 per group

Next, we investigated the downstream target genes of miR-374a-5p. We found that expression of *ARNT*, *BMP2*, *CRISPLD1*, *FGFR2*, *JARID2*, *MSX1*, *NOG*, *RUNX2*, *WNT5A*, and *ZNF236* was significantly downregulated in cultured cells treated with miR*-*374a-5p mimic (Fig. 4a). By contrast, a miR-374a-5p inhibitor induced the expression of *CRISPLD1*, *FGFR2*, *JARID2*, *MSX1*, *TNS1*, and *ZNF236* (Fig. 4b). Therefore, these results indicate that miR-374a-5p can regulate the expression of *CRISPLD1*, *FGFR2*, *JARID2*, *MSX1*, and *ZNF236* in a dose-dependent manner in cultured human palate cells.

**Fig. 5:**
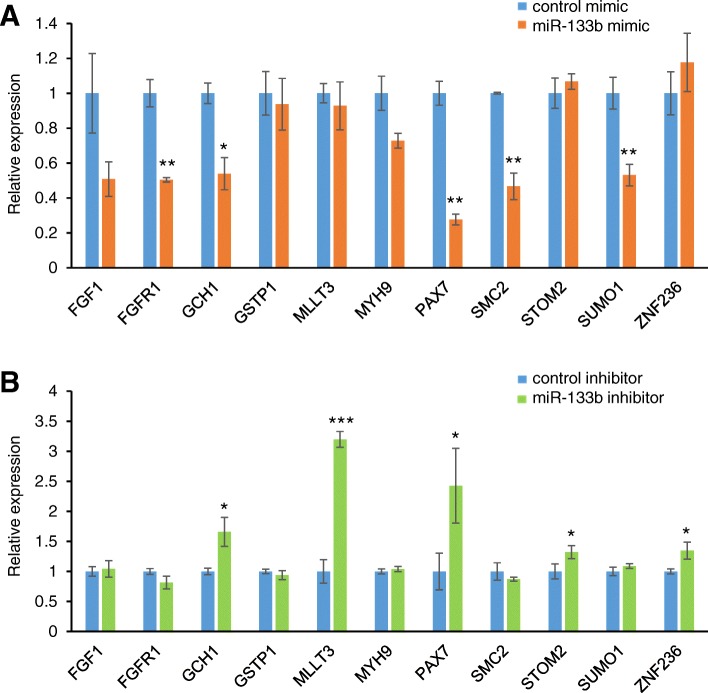
Effect of miR-133b on predicted target genes. **a** Quantitative RT-PCR for the indicated genes after treatment with negative control (light blue) or miR-133b mimic (orange). * *p* < 0.05, ** *p* < 0.01. Each treatment group was compared with the control. n = 6 per group. **b** Quantitative RT-PCR for the indicated genes after treatment with negative control (light blue) or miR-133b inhibitor (light green). * *p* < 0.05, *** *p* < 0.001. Each treatment group was compared with the control. n = 6 per group

Lastly, we assessed the predicted miR-133b downstream target genes. We found that expression of *FGFR1*, *GCH1, PAX7*, *SMC2*, and *SUMO1* was significantly downregulated in cultured cells treated with miR-133b mimic (Fig. 5a), but expression of *GCH1*, *MLLT3*, *PAX7*, *STOM2* and *ZNF236* was significantly increased with a miR-133b inhibitor (Fig. 5b). These results indicate that miR-133b can regulate the expression of *GCH1* and *PAX7* in a dose-dependent manner in cultured human palate cells. Taken together, our experimental results provide proof of function for some of the predicted target genes (*ERBB2* and *MTHFD1* for miR-4680-3p; *CRISPLD1*, *FGFR2*, *JARID2*, *MSX1*, and *ZNF236* for miR-374a-5p; and *GCH1* and *PAX7* for miR-133b) in cultured human palate cells.

## Discussion

CP-associated genes were grouped based on their common features through GO and KEGG analyses. As expected, most of the pathways highlighted have been shown to be involved in the growth and development process. For example, in the top enriched pathways, the MAPK pathway regulated by growth factors (e.g. hedgehog, TGFβ, and WNT) can regulate a wide variety of cellular functions crucial for palatogenesis, including cell proliferation and differentiation [22]. The GO term annotation showed that the transcription process is the most significantly enriched (67%). This suggests that transcription factors regulated by cellular pathways that control the growth and fusion of the palatal shelves are crucial for palate development. For example, loss of TGFβ receptor type II (*Tgfbr2*) results in ectopic p38 MAPK activation and altered gene expression of *Adcy2* and *Pde4b*, which regulate lipid metabolism and cause CP in mice [23]. In the enriched cellular component terms, we identified a focus on membranes and other structures dependent on lipids and lipid bilayers for their structure and function. Six genes in the CP gene list were involved in the cilium: *GLI2*, *GLI3*, *KIF7*, *OFD1*, *PAFAH1B1,* and *WDR19*. GLI2 and GLI3 locate in the primary cilium and translocate into the nuclei upon binding of hedgehog ligands to activate and/or inactivate hedgehog signaling [24, 25]. KIF7 is a motor protein in all cilia that regulates hedgehog signaling [26–28], and OFD1 and WDR19 localize around the basal body at the base of cilia [29–31]. PAFAH1B1 is a regulator of the dynein motor proteins that traffic molecules back down the cilium [32–34]. Thus, the accumulating evidences indicate that primary cilia contain abundant hedgehog receptors and mediators, and that they regulate hedgehog signaling activity.

In non-syndromic CP, maternal environmental factors most likely increase the risk of CP with a link to some single-nucleotide polymorphisms (SNPs), while these SNPs alone do not achieve genome-wide significance. For example, SNPs in *GSTP1*, *TBK1* and *ZNF236* seem to be associated with a higher risk of CP with maternal smoking [35, 36]. Similarly, SNPs in *MLLT3* and *SMC2* seem to increase CP risk with alcohol consumption during the peri-conceptual period [35]. Importantly, smoking and alcohol consumption, which are associated not only with cancer but also with other diseases, alter miRNA expression in the serum and cells [37–41]. During development, maternal alcohol consumption directly influences miRNA expression in mice and zebrafish [42–44]. Recent studies suggest that miRNAs may pass through the placenta from mothers to embryos to directly regulate embryogenesis [45, 46]. In this study, we found that some CP genes are regulated by multiple miRNAs, two miRNAs for *GSTP1*, 26 miRNAs for *MLLT3*, 29 miRNAs for *SMC2*, 22 miRNAs for *TBK1*, and 56 miRNAs for *ZNF236*. These CP genes may have a higher chance of being altered by environmental factors.

## Conclusions

Our computational analyses have predicted the possible roles and mechanisms of miRNAs altered by environmental factors in CP. Overexpression of miR-374a, miR-4680, and miR-133b suppresses cell proliferation through the regulation of their target genes in cultured HEPM cells. While this systematic review shows much strength in the collection of CP-associated genes, it presents some limitations in the identification of causative genes due to the complex etiology of CP (e.g. genes not specific to CP, CP that is a part of syndromic features, no complete penetrance, secondary CP affected by other craniofacial anomalies).

## Additional files

Additional file 1: **Table S1.** PCR primer sets used in this study. (XLSX 12 kb)

Additional file 2: **Table S2.** Gene mutations found in cases of human CP. (PDF 704 kb)

Additional file 3: **Table S3.** Genes with significant contribution to human cleft palate. (XLSX 89 kb)

Additional file 4: **Table S4.** Genes without significant contribution to human cleft palate. (XLSX 38 kb)

Additional file 5: **Table S5.** KEGG pathways enriched with human cleft palate genes. (XLSX 14 kb)

Additional file 6: **Table S6.** GO terms enriched with human cleft palate genes. (XLSX 19 kb)

Additional file 7: **Table S7.** MicroRNA enrichment analysis of human cleft palate genes. (XLSX 9 kb)

Additional file 8: **Table S8.** Transfection efficiency of miRNA mimic and inhibitor. (PDF 51 kb)

## Data Availability

All the data from this study are available as supplemental information.

## Abbreviations

CL/CP: Cleft lip with/without cleft palate
CLO: Cleft lip only
CLP: Cleft lip with cleft palate
CNC: Cranial neural crest
CPO: Cleft palate only
miRNA: MicroRNA
