# Supplementary material for: MicroRNA-374a, -4680, and -133b suppress cell proliferation through the regulation of genes associated with human cleft palate in cultured human palate cells
Source: BMC Med Genomics. 2019 Jul 1;12:93. doi: 10.1186/s12920-019-0546-z (PMC6604454; doi:10.1186/s12920-019-0546-z)
Supplement: Supplementary file 2 — Table S2. Gene mutations found in cases of human CP. (PDF 704 kb) [file 12920_2019_546_MOESM2_ESM.pdf]

Table S2. Gene mutations found in cases of human CP

No	Gene symbol	Gene name	Reference	Syndrome
1	*ACTB*	actin, beta	[1]	Baraitser-Winter cerebrofrontofacial syndrome
2	*ACTG1*	actin, gamma 1	[1]	Baraitser-Winter cerebrofrontofacial syndrome
3	*ALX1*	ALX homeobox 1	[2]	Frontofacionasal dysplasia
4	*ALX3*	ALX homeobox 3	[3]	Autosomal recessive frontorhiny
5	*ANKRD11*	Ankyrin repeat domain 11	[4]	KBG syndrome
6	*B3GALTL*	Beta 1,3-galactosyltransferase-like	[5]	Peters plus syndrome
7	*BCOR*	BCL6 co-repressor	[6]	Oculofaciocardiodental syndrome
8	*BMP4*	Bone morphogenetic protein 4	[7]	Non-syndromic CL/CP
9	*CDH1*	Cadherin 1, type 1, E-cadherin (epithelial)	[8]	Familial gastric cancer and CLP
10	*CHD7*	Chromodomain helicase DNA binding protein 7	[9]	CHARGE syndrome
11	*CHRNG*	Cholinergic receptor, nicotinic, gamma	[10]	Lethal and Escobar multiple pterygium syndrome
12	*COL2A1*	Collagen, type II, alpha 1	[11]	Stickler syndrome, type 1
13	*COL11A1*	Collagen, type XI, alpha 1	[12]	Stickler syndrome, type 2
14	*COL11A2*	Collagen, type XI, alpha 2	[13]	Stickler syndrome, type 3
15	*CRISPLD2*	Cysteine-rich secretory protein LCCL domain containing 2	[14]	Non-syndromic CL/CP
16	*CYP1A1*	Cytochrome P450, family 1, subfamily A, polypeptide 1	[15]	Non-syndromic CL/CP
17	*DHCR7*	7-dehydrocholesterol reductase	[16]	Smith-Lemli-Opitz syndrome
18	*DHCR24*	24-dehydrocholesterol reductase	[17-19]	Desmosterolosis
19	*DHODH*	Dihydroorotate dehydrogenase (quinone)	[20]	Miller syndrome
20	*DOK7*	Docking protein 7	[21]	Fetal akinesia deformation sequence syndrome
21	*EFNB1*	Ephrin-B1	[22]	Craniofrontonasal syndrome
22	*EFTUD2*	Elongation Factor Tu GTP Binding Domain Containing 2	[23]	Mandibulofacial dysostosis with microcephaly
23	*ESCO2*	Establishment of cohesion 1 homolog 2 (S. cerevisiae)	[24]	Roberts syndrome
24	*EYA1*	Eyes absent homolog 1 (Drosophila)	[25]	Stickler and branchio-oto-renal syndrome
25	*FAM20C*	Family with sequence similarity 20, member C	[26, 27]	Raine syndrome
26	*FGF1*	Fibroblast growth factor 1 (acidic)	[28]	Non-syndromic CL/CP
27	*FGF8*	Fibroblast growth factor 8 (androgen-induced)	[29]	Kallmann syndrome
28	*FGF10*	Fibroblast growth factor 10	[30]	Non-syndromic CL/CP
29	*FGFR1*	Fibroblast growth factor receptor 1	[31]	Kallmann syndrome
30	*FGFR2*	Fibroblast growth factor receptor 2	[32, 33]	Crouzon syndrome & Apert syndrome
31	*FLNA*	Filamin A, alpha	[34, 35]	Otopalatodigital syndrome types 1 and 2
32	*FLNB*	Filamin B, beta	[36-38]	Larsen syndrome & atelosteogenesis

33	*FOXC2*	Forkhead box C2 (MFH-1, mesenchyme forkhead 1)	[39, 40]	Hereditary lymphedema-distichiasis syndrome
34	*FOXE1*	Forkhead box E1 (thyroid transcription factor 2)	[41-43]	Bamforth-Lazarus syndrome
35	*FRAS1*	Fraser syndrome 1	[44, 45]	Fraser syndrome
36	*FREM1*	FRAS1-related extracellular matrix protein 1	[46]	Bifid nose, renal agenesis, and anorectal malformations syndrome
37	*FREM2*	FRAS1-related extracellular matrix protein 2	[44, 45]	Fraser syndrome
38	*FZD4*	Frizzled class receptor 4	[47-49]	Autosomal dominant exudative vitreoretinopathy
39	*GABRB3*	gamma-aminobutyric (GABA) A receptor, beta 3	[50]	Non-syndromic CL/CP
40	*GDF6*	Growth differentiation factor 6	[51]	Non-syndromic CL/CP
41	*GLI2*	GLI family zinc finger 2	[52-54]	Holoprosencephaly
42	*GLI3*	GLI family zinc finger 3	[55]	Oro-facial-digital syndrome
43	*GPC3*	Glypican 3	[56, 57]	Simpson-Golabi-Behmel syndrome
44	*GRHL3*	Grainyhead-like 3	[58]	Van der Woude syndrome
45	*GSTT1*	Glutathione S-transferase theta 1	[15]	Non-syndromic CL/CP
46	*HIC1*	Hypermethylated in cancer 1	[59]	Miller-Dieker syndrome
47	*HYLS1*	Hydrolethalus syndrome 1	[60]	Hydrolethalus syndrome
48	*IRF6*	Interferon regulatory factor 6	[61-63]	Van der Woude syndrome/ Popliteal pterygium syndrome/ Non-syndromic CLP
49	*JAG2*	Jagged 2	[64, 65]	Non-syndromic CL/CP
50	*KAT6B (aka MYST4)*	K(Lysine) Acetyltransferase 6B	[66, 67]	Say-Barber-Biesecker-Young-Simpson syndrome
51	*KCNJ2*	Potassium inwardly-rectifying channel, subfamily J, member 2	[68, 69]	Andersen syndrome
52	*KDM1A*	Lysine (K)-specific demethylase 1A	[4]	KBG syndrome, Kabuki syndrome
53	*KDM6A*	Lysine (K)-specific demethylase 6A	[70]	Kabuki syndrome
54	*KIF7*	Kinesin family member 7	[71]	Hydrolethalus and acrocallosal syndrome
55	*KMT2D (aka MLL2)*	Lysine (K)-specific methyltransferase 2D	[72]	Kabuki syndrome
56	*LMX1B*	LIM homeobox transcription factor 1, beta	[73]	Nail-patella syndrome (hereditary onycho-osteodystrophy)
57	*MAFB*	v-maf avian musculoaponeurotic fibrosarcome oncogene homolog B	[74, 75]	Non-syndromic
58	*MEOX1*	Mesenchyme homeobox 1	[76]	Klippel-Feil syndrome
59	*MID1*	Midline 1 (Opitz/BBB syndrome)	[77, 78]	Opitz G/BBB syndrome
60	*MSX1*	msh homeobox 1	[79]	Non-syndromic CL/CP
61	*MTHFR*	Methlenetetrahydrofolate reductase (NAD(P)H)	[80, 81]	Non-syndromic CL/CP
62	*MYH9*	Myosin, heavy chain 9, non-muscle	[51, 82]	Non-syndromic CL/CP
63	*NAT2*	N-acetyltransferase 2 (arylamine N-acetyltransferase)	[15]	Non-syndromic CL/CP
64	*NBN*	Nibrin	[83]	Nijmegen breakage syndrome

65	*NIPBL*	Nipped-B homolog (Drosophila)	[84, 85]	Cornelia de Lange syndrome
66	*OFD1*	Oral-facial-digital syndrome 1	[86]	Oral-facial-digital syndrome 1
67	*OTX2*	Orthodenticle homeobox 2	[87]	Hemifacial microsomia
68	*PAFAH1B1*	Platelet-activating factor acetylhydrolase, isoform Ib, subunit 1 (45kDa)	[88]	Miller-Dieker syndrome
69	*PAX7*	Paired box 7	[89]	Non-syndromic CL/CP
70	*PAX9*	Paired box 9	[90]	Non-syndromic CL/CP
71	*PDGFC*	Platelet derived growth factor C	[91]	Non-syndromic CL/CP
72	*PHF8*	PHD finger protein 8	[92]	X-linked mental retardation and CL/CP
73	*PQBP1*	Polyglutamine binding protein 1	[93]	X-linked mental retardation
74	*PRICKLE1*	Prickle homolog 1 (Drosophila)	[94]	Velocardiofacial syndrome
75	*PTCH1*	Patched homolog 1 (Drosophila)	[95, 96]	Gorlin syndrome
76	*PVRL1*	Poliovirus receptor-related 1 (herpesvirus entry mediator C)	[97, 98]	CLP - ectodermal dysplasia
77	*RECQL4*	RecQ protein-like 4	[93]	Rothmund-Thomson syndrome, RAPADILINO syndrome
78	*ROR2*	Receptor tyrosine kinase-like orphan receptor 2	[99]	Robinow syndrome
79	*SATB2*	SATB homeobox 2	[40, 65, 100]	Isolated cleft palate
80	*SDC2*	Syndecan 2	[51]	Non-syndromic CL/CP
81	*SF3B4*	Splicing factor 3b, subunit 4, 49kDa	[101]	Nager syndrome
82	*SIX3*	SIX homeobox 3	[102]	Holoprosencephaly
83	*SKI*	v-ski avian sarcoma viral oncogene homolog	[65, 103]	Non-syndromic CL/CP
84	*SLC26A2*	Solute carrier family 26 (sulfate transporter), member 2	[104]	Diastrophic dysplasia
85	*SOX9*	SRY (sex determining region Y)-box 9	[105-108]	Campomelic dysplasia & Pierre Robin syndrome
86	*SOX11*	SRY (sex determining region Y)-box 11	[109]	Opit "C" trigonocephaly syndrome
87	*SUMO1*	Small ubiquitin-like modifier 1	[110, 111]	Non-syndromic CL/CP
88	*TBX1*	T-box 1	[112-114]	DiGeorge syndrome
89	*TBX22*	T-box 22	[115-118]	X-linked cleft palate and ankyloglossia
90	*TCOF1*	Treacher Collins-Franceschetti syndrome 1	[119]	Treacher Collins syndrome
91	*TFAP2A*	Transcription factor AP-2 alpha (activating enhancer binding protein 2 alpha)	[120]	Branchio-oculo-facial syndrome
92	*TGFA*	Transforming growth factor, alpha	[121-123]	Non-syndromic CL/P
93	*TGFB3*	Transforming growth factor, beta 3	[124]	Non-syndromic CL/P
94	*TGFBR1*	Transforming growth factor, beta receptor 1	[125]	Loeys-Dietz syndrome
95	*TGFBR2*	Transforming growth factor, beta receptor II (70/80kDa)	[125]	Loeys-Dietz syndrome
96	*TGIF1*	TGFB-induced factor homeobox 1	[126]	Holoprosencephaly
97	*TIMP2*	Tissue inhibitor of metalloproteinase 2	[28]	Non-syndromic CL/CP

98	*TP63*	Tumor protein p63	[127-133]	Hay-Wells syndrome, Ectrodactyly, ectodermal dysplasia and cleft lip/palate (EEC) syndrome, Ankyloblepharon-ectodermal dysplasia-clefting syndrome
99	*TWIST1*	twist homolog 1 (Drosophila)	[134, 135]	Saethre-Chotzen syndrome
100	*UBB*	Ubiquitin B precursor	[136]	Smith-Magenis syndrome
101	*VAX1*	Ventral anterior homeobox 1	[89, 137]	Non-syndromic CL/CP
102	*WDR19 (aka IFT144)*	WD repeat domain 19	[138, 139]	Ciliopathies with skeletal anomalies and renal insufficiency
103	*WHSC1*	Wolf-Hirschhorn syndrome candidate 1	[140]	Wolf-Hirschhorn syndrome
104	*WNT3*	wingless-type MMTV integration site family, member 3	[141]	Tetra-amelia with CLP & Non-syndromic CL/CP
105	*WNT3A*	wingless-type MMTV integration site family, member 3A	[142]	Non-syndromic CL/CP
106	*WNT5A*	wingless-type MMTV integration site family, member 5A	[142]	Non-syndromic CL/CP
107	*WNT11*	wingless-type MMTV integration site family, member 11	[142]	Non-syndromic CL/CP
108	*WTX (aka FAM123B)*	Wilms tumor on the X-chromosome	[143, 144]	Osteopathia striata with cranial sclerosis
109	*YWHAE*	Tyrosine 3-monooxygenase/tryptophan 5-monooxygenase activation protein, epsilon polypeptide	[88, 145]	Miller-Dieker syndrome
110	*ZEB2*	Zinc finger E-box binding homeobox 2	[146]	Mowat-Wilson syndrome

CLP, cleft lip and palate; CL/CP, cleft lip with or without cleft palate.

References

1. Di Donato N, Rump A, Koenig R, Der Kaloustian VM, Halal F, Sonntag K, Krause C, Hackmann K, Hahn G, Schrock E *et al*: **Severe forms of Baraitser-Winter syndrome are caused by ACTB mutations rather than ACTG1 mutations**. *European journal of human genetics : EJHG* 2014, **22**(2):179-183.
2. Uz E, Alanay Y, Aktas D, Vargel I, Gucer S, Tuncbilek G, von Eggeling F, Yilmaz E, Deren O, Posorski N *et al*: **Disruption of ALX1 causes extreme microphthalmia and severe facial clefting: expanding the spectrum of autosomal-recessive ALX-related frontonasal dysplasia**. *Am J Hum Genet* 2010, **86**(5):789-796.
3. Twigg SR, Versnel SL, Nurnberg G, Lees MM, Bhat M, Hammond P, Hennekam RC, Hoogeboom AJ, Hurst JA, Johnson D *et al*: **Frontorhiny, a distinctive presentation of frontonasal dysplasia caused by recessive mutations in the ALX3 homeobox gene**. *Am J Hum Genet* 2009, **84**(5):698-705.
4. Tunovic S, Barkovich J, Sherr EH, Slavotinek AM: **De novo ANKRD11 and KDM1A gene mutations in a male with features of KBG syndrome and Kabuki syndrome**. *Am J Med Genet A* 2014, **164A**(7):1744-1749.
5. Lesnik Oberstein SA, Kriek M, White SJ, Kalf ME, Szuhai K, den Dunnen JT, Breuning MH, Hennekam RC: **Peters Plus syndrome is caused by mutations in B3GALTL, a putative glycosyltransferase**. *Am J Hum Genet* 2006, **79**(3):562-566.
6. Ng D, Thakker N, Corcoran CM, Donnai D, Perveen R, Schneider A, Hadley DW, Tifft C, Zhang L, Wilkie AO *et al*: **Oculofaciocardiodental and Lenz microphthalmia syndromes result from distinct classes of mutations in BCOR**. *Nat Genet* 2004, **36**(4):411-416.
7. Suzuki S, Marazita ML, Cooper ME, Miwa N, Hing A, Jugessur A, Natsume N, Shimozato K, Ohbayashi N, Suzuki Y *et al*: **Mutations in BMP4 are associated with subepithelial, microform, and overt cleft lip**. *Am J Hum Genet* 2009, **84**(3):406-411.
8. Frebourg T, Oliveira C, Hochain P, Karam R, Manouvrier S, Graziadio C, Vekemans M, Hartmann A, Baert-Desurmont S, Alexandre C *et al*: **Cleft lip/palate and CDH1/E-cadherin mutations in families with hereditary diffuse gastric cancer**. *J Med Genet* 2006, **43**(2):138-142.
9. Vissers LE, van Ravenswaaij CM, Admiraal R, Hurst JA, de Vries BB, Janssen IM, van der Vliet WA, Huys EH, de Jong PJ, Hamel BC *et al*: **Mutations in a new member of the chromodomain gene family cause CHARGE syndrome**. *Nat Genet* 2004, **36**(9):955-957.
10. Morgan NV, Brueton LA, Cox P, Greally MT, Tolmie J, Pasha S, Aligianis IA, van Bokhoven H, Marton T, Al-Gazali L *et al*: **Mutations in the embryonal subunit of the acetylcholine receptor (CHRNG) cause lethal and Escobar variants of multiple pterygium syndrome**. *Am J Hum Genet* 2006, **79**(2):390-395.
11. Snead MP, Payne SJ, Barton DE, Yates JR, al-Imara L, Pope FM, Scott JD: **Stickler syndrome: correlation between vitreoretinal phenotypes and linkage to COL 2A1**. *Eye* 1994, **8 (Pt 6)**:609-614.
12. Richards AJ, Yates JR, Williams R, Payne SJ, Pope FM, Scott JD, Snead MP: **A family with Stickler syndrome type 2 has a mutation in the COL11A1 gene resulting in the substitution of glycine 97 by valine in alpha 1 (XI) collagen**. *Hum Mol Genet* 1996, **5**(9):1339-1343.
13. Vikkula M, Mariman EC, Lui VC, Zhidkova NI, Tiller GE, Goldring MB, van Beersum SE, de Waal Malefijt MC, van den Hoogen FH, Ropers HH *et al*: **Autosomal dominant and recessive osteochondrodysplasias associated with the COL11A2 locus**. *Cell* 1995, **80**(3):431-437.
14. Chiquet BT, Lidral AC, Stal S, Mulliken JB, Moreno LM, Arcos-Burgos M, Valencia-Ramirez C, Blanton SH, Hecht JT: **CRISPLD2: a novel NSCLP candidate gene**. *Hum Mol Genet* 2007, **16**(18):2241-2248.

15. Shi M, Christensen K, Weinberg CR, Romitti P, Bathum L, Lozada A, Morris RW, Lovett M, Murray JC: **Orofacial cleft risk is increased with maternal smoking and specific detoxification-gene variants**. *Am J Hum Genet* 2007, **80**(1):76-90.
16. Wassif CA, Maslen C, Kachilele-Linjewile S, Lin D, Linck LM, Connor WE, Steiner RD, Porter FD: **Mutations in the human sterol delta7-reductase gene at 11q12-13 cause Smith-Lemli-Opitz syndrome**. *Am J Hum Genet* 1998, **63**(1):55-62.
17. Andersson HC, Kratz L, Kelley R: **Desmosterolosis presenting with multiple congenital anomalies and profound developmental delay**. *American journal of medical genetics* 2002, **113**(4):315-319.
18. FitzPatrick DR, Keeling JW, Evans MJ, Kan AE, Bell JE, Porteous ME, Mills K, Winter RM, Clayton PT: **Clinical phenotype of desmosterolosis**. *American journal of medical genetics* 1998, **75**(2):145-152.
19. Waterham HR, Koster J, Romeijn GJ, Hennekam RC, Vreken P, Andersson HC, FitzPatrick DR, Kelley RI, Wanders RJ: **Mutations in the 3beta-hydroxysterol Delta24-reductase gene cause desmosterolosis, an autosomal recessive disorder of cholesterol biosynthesis**. *Am J Hum Genet* 2001, **69**(4):685-694.
20. Ng SB, Buckingham KJ, Lee C, Bigham AW, Tabor HK, Dent KM, Huff CD, Shannon PT, Jabs EW, Nickerson DA *et al*: **Exome sequencing identifies the cause of a mendelian disorder**. *Nat Genet* 2010, **42**(1):30-35.
21. Vogt J, Morgan NV, Marton T, Maxwell S, Harrison BJ, Beeson D, Maher ER: **Germline mutation in DOK7 associated with fetal akinesia deformation sequence**. *J Med Genet* 2009, **46**(5):338-340.
22. Twigg SR, Kan R, Babbs C, Bochukova EG, Robertson SP, Wall SA, Morriss-Kay GM, Wilkie AO: **Mutations of ephrin-B1 (EFNB1), a marker of tissue boundary formation, cause craniofrontonasal syndrome**. *Proc Natl Acad Sci U S A* 2004, **101**(23):8652-8657.
23. Lines MA, Huang L, Schwartzentruber J, Douglas SL, Lynch DC, Beaulieu C, Guion-Almeida ML, Zechi-Ceide RM, Gener B, Gillessen-Kaesbach G *et al*: **Haploinsufficiency of a spliceosomal GTPase encoded by EFTUD2 causes mandibulofacial dysostosis with microcephaly**. *Am J Hum Genet* 2012, **90**(2):369-377.
24. Vega H, Waisfisz Q, Gordillo M, Sakai N, Yanagihara I, Yamada M, van Gosliga D, Kayserili H, Xu C, Ozono K *et al*: **Roberts syndrome is caused by mutations in ESCO2, a human homolog of yeast ECO1 that is essential for the establishment of sister chromatid cohesion**. *Nat Genet* 2005, **37**(5):468-470.
25. Olavarrieta L, Morales-Angulo C, del Castillo I, Moreno F, Moreno-Pelayo MA: **Stickler and branchio-oto-renal syndromes in a patient with mutations in EYA1 and COL2A1 genes**. *Clin Genet* 2008, **73**(3):262-267.
26. Raine J, Winter RM, Davey A, Tucker SM: **Unknown syndrome: microcephaly, hypoplastic nose, exophthalmos, gum hyperplasia, cleft palate, low set ears, and osteosclerosis**. *J Med Genet* 1989, **26**(12):786-788.
27. Simpson MA, Hsu R, Keir LS, Hao J, Sivapalan G, Ernst LM, Zackai EH, Al-Gazali LI, Hulskamp G, Kingston HM *et al*: **Mutations in FAM20C are associated with lethal osteosclerotic bone dysplasia (Raine syndrome), highlighting a crucial molecule in bone development**. *Am J Hum Genet* 2007, **81**(5):906-912.
28. Nikopensius T, Kempa I, Ambrozaityte L, Jagomagi T, Saag M, Matuleviciene A, Utkus A, Krjutskov K, Tammekivi V, Piekuse L *et al*: **Variation in FGF1, FOXE1, and TIMP2 genes is associated with nonsyndromic cleft lip with or without cleft palate**. *Birth Defects Res A Clin Mol Teratol* 2011, **91**(4):218-225.
29. Falardeau J, Chung WC, Beenken A, Raivio T, Plummer L, Sidis Y, Jacobson-Dickman EE, Eliseenkova AV, Ma J, Dwyer A *et al*: **Decreased FGF8 signaling causes deficiency of**

gonadotropin-releasing hormone in humans and mice. *The Journal of clinical investigation* 2008, **118**(8):2822-2831.

30. Riley BM, Mansilla MA, Ma J, Daack-Hirsch S, Maher BS, Raffensperger LM, Russo ET, Vieira AR, Dode C, Mohammadi M *et al*: **Impaired FGF signaling contributes to cleft lip and palate**. *Proc Natl Acad Sci U S A* 2007, **104**(11):4512-4517.
31. Dode C, Levilliers J, Dupont JM, De Paepe A, Le Du N, Soussi-Yanicostas N, Coimbra RS, Delmaghani S, Compain-Nouaille S, Baverel F *et al*: **Loss-of-function mutations in FGFR1 cause autosomal dominant Kallmann syndrome**. *Nat Genet* 2003, **33**(4):463-465.
32. Reardon W, Winter RM, Rutland P, Pulleyn LJ, Jones BM, Malcolm S: **Mutations in the fibroblast growth factor receptor 2 gene cause Crouzon syndrome**. *Nat Genet* 1994, **8**(1):98-103.
33. Wilkie AO, Slaney SF, Oldridge M, Poole MD, Ashworth GJ, Hockley AD, Hayward RD, David DJ, Pulleyn LJ, Rutland P *et al*: **Apert syndrome results from localized mutations of FGFR2 and is allelic with Crouzon syndrome**. *Nat Genet* 1995, **9**(2):165-172.
34. Robertson SP, Twigg SR, Sutherland-Smith AJ, Biancalana V, Gorlin RJ, Horn D, Kenwrick SJ, Kim CA, Morava E, Newbury-Ecob R *et al*: **Localized mutations in the gene encoding the cytoskeletal protein filamin A cause diverse malformations in humans**. *Nat Genet* 2003, **33**(4):487-491.
35. Hehr U, Hehr A, Uyanik G, Phelan E, Winkler J, Reardon W: **A filamin A splice mutation resulting in a syndrome of facial dysmorphism, periventricular nodular heterotopia, and severe constipation reminiscent of cerebro-fronto-facial syndrome**. *J Med Genet* 2006, **43**(6):541-544.
36. Krakow D, Robertson SP, King LM, Morgan T, Sebald ET, Bertolotto C, Wachsmann-Hogiu S, Acuna D, Shapiro SS, Takafuta T *et al*: **Mutations in the gene encoding filamin B disrupt vertebral segmentation, joint formation and skeletogenesis**. *Nat Genet* 2004, **36**(4):405-410.
37. Zhang D, Herring JA, Swaney SS, McClendon TB, Gao X, Browne RH, Rathjen KE, Johnston CE, Harris S, Cain NM *et al*: **Mutations responsible for Larsen syndrome cluster in the FLNB protein**. *J Med Genet* 2006, **43**(5):e24.
38. Bicknell LS, Farrington-Rock C, Shafeghati Y, Rump P, Alanay Y, Alembik Y, Al-Madani N, Firth H, Karimi-Nejad MH, Kim CA *et al*: **A molecular and clinical study of Larsen syndrome caused by mutations in FLNB**. *J Med Genet* 2007, **44**(2):89-98.
39. Fang J, Dagenais SL, Erickson RP, Arlt MF, Glynn MW, Gorski JL, Seaver LH, Glover TW: **Mutations in FOXC2 (MFH-1), a forkhead family transcription factor, are responsible for the hereditary lymphedema-distichiasis syndrome**. *Am J Hum Genet* 2000, **67**(6):1382-1388.
40. Wilkie AO, Morriss-Kay GM: **Genetics of craniofacial development and malformation**. *Nat Rev Genet* 2001, **2**(6):458-468.
41. Clifton-Bligh RJ, Wentworth JM, Heinz P, Crisp MS, John R, Lazarus JH, Ludgate M, Chatterjee VK: **Mutation of the gene encoding human TTF-2 associated with thyroid agenesis, cleft palate and choanal atresia**. *Nat Genet* 1998, **19**(4):399-401.
42. Castanet M, Park SM, Smith A, Bost M, Leger J, Lyonnet S, Pelet A, Czernichow P, Chatterjee K, Polak M: **A novel loss-of-function mutation in TTF-2 is associated with congenital hypothyroidism, thyroid agenesis and cleft palate**. *Hum Mol Genet* 2002, **11**(17):2051-2059.
43. Moreno LM, Mansilla MA, Bullard SA, Cooper ME, Busch TD, Machida J, Johnson MK, Brauer D, Krahn K, Daack-Hirsch S *et al*: **FOXE1 association with both isolated cleft lip with or without cleft palate, and isolated cleft palate**. *Hum Mol Genet* 2009, **18**(24):4879-4896.
44. Jadeja S, Smyth I, Pitera JE, Taylor MS, van Haelst M, Bentley E, McGregor L, Hopkins J, Chalepakis G, Philip N *et al*: **Identification of a new gene mutated in Fraser syndrome and mouse myelencephalic blebs**. *Nat Genet* 2005, **37**(5):520-525.
45. Slavotinek AM, Tifft CJ: **Fraser syndrome and cryptophthalmos: review of the diagnostic criteria and evidence for phenotypic modules in complex malformation syndromes**. *J Med Genet* 2002, **39**(9):623-633.

46. Alazami AM, Shaheen R, Alzahrani F, Snape K, Saggar A, Brinkmann B, Bavi P, Al-Gazali LI, Alkuraya FS: **FREM1 mutations cause bifid nose, renal agenesis, and anorectal malformations syndrome**. *Am J Hum Genet* 2009, **85**(3):414-418.
47. Li P, Zhang HZ, Huff S, Nimmakayalu M, Qumsiyeh M, Yu J, Szekely A, Xu T, Pober BR: **Karyotype-phenotype insights from 11q14.1-q23.2 interstitial deletions: FZD4 haploinsufficiency and exudative vitreoretinopathy in a patient with a complex chromosome rearrangement**. *Am J Med Genet A* 2006, **140**(24):2721-2729.
48. Robitaille J, MacDonald ML, Kaykas A, Sheldahl LC, Zeisler J, Dube MP, Zhang LH, Singaraja RR, Guernsey DL, Zheng B *et al*: **Mutant frizzled-4 disrupts retinal angiogenesis in familial exudative vitreoretinopathy**. *Nat Genet* 2002, **32**(2):326-330.
49. Toomes C, Bottomley HM, Scott S, Mackey DA, Craig JE, Appukuttan B, Stout JT, Flaxel CJ, Zhang K, Black GC *et al*: **Spectrum and frequency of FZD4 mutations in familial exudative vitreoretinopathy**. *Investigative ophthalmology & visual science* 2004, **45**(7):2083-2090.
50. Scapoli L, Martinelli M, Pezzetti F, Carinci F, Bodo M, Tognon M, Carinci P: **Linkage disequilibrium between GABRB3 gene and nonsyndromic familial cleft lip with or without cleft palate**. *Human genetics* 2002, **110**(1):15-20.
51. Chiquet BT, Hashmi SS, Henry R, Burt A, Mulliken JB, Stal S, Bray M, Blanton SH, Hecht JT: **Genomic screening identifies novel linkages and provides further evidence for a role of MYH9 in nonsyndromic cleft lip and palate**. *European journal of human genetics : EJHG* 2009, **17**(2):195-204.
52. Bertolacini CD, Ribeiro-Bicudo LA, Petrin A, Richieri-Costa A, Murray JC: **Clinical findings in patients with GLI2 mutations--phenotypic variability**. *Clinical genetics* 2012, **81**(1):70-75.
53. Kevelam SH, van Harssel JJ, van der Zwaag B, Smeets HJ, Paulussen AD, Lichtenbelt KD: **A patient with a mild holoprosencephaly spectrum phenotype and heterotaxy and a 1.3 Mb deletion encompassing GLI2**. *Am J Med Genet A* 2012, **158A**(1):166-173.
54. Roessler E, Du YZ, Mullor JL, Casas E, Allen WP, Gillessen-Kaesbach G, Roeder ER, Ming JE, Ruiz i Altaba A, Muenke M: **Loss-of-function mutations in the human GLI2 gene are associated with pituitary anomalies and holoprosencephaly-like features**. *Proceedings of the National Academy of Sciences of the United States of America* 2003, **100**(23):13424-13429.
55. Johnston JJ, Sapp JC, Turner JT, Amor D, Aftimos S, Aleck KA, Bocian M, Bodurtha JN, Cox GF, Curry CJ *et al*: **Molecular analysis expands the spectrum of phenotypes associated with GLI3 mutations**. *Human mutation* 2010, **31**(10):1142-1154.
56. Li M, Shuman C, Fei YL, Cutiongco E, Bender HA, Stevens C, Wilkins-Haug L, Day-Salvatore D, Yong SL, Geraghty MT *et al*: **GPC3 mutation analysis in a spectrum of patients with overgrowth expands the phenotype of Simpson-Golabi-Behmel syndrome**. *American journal of medical genetics* 2001, **102**(2):161-168.
57. Pilia G, Hughes-Benzie RM, MacKenzie A, Baybayan P, Chen EY, Huber R, Neri G, Cao A, Forabosco A, Schlessinger D: **Mutations in GPC3, a glypican gene, cause the Simpson-Golabi-Behmel overgrowth syndrome**. *Nat Genet* 1996, **12**(3):241-247.
58. Peyrard-Janvid M, Leslie EJ, Kousa YA, Smith TL, Dunnwald M, Magnusson M, Lentz BA, Unneberg P, Fransson I, Koillinen HK *et al*: **Dominant mutations in GRHL3 cause Van der Woude Syndrome and disrupt oral periderm development**. *American journal of human genetics* 2014, **94**(1):23-32.
59. Carter MG, Johns MA, Zeng X, Zhou L, Zink MC, Mankowski JL, Donovan DM, Baylin SB: **Mice deficient in the candidate tumor suppressor gene Hic1 exhibit developmental defects of structures affected in the Miller-Dieker syndrome**. *Hum Mol Genet* 2000, **9**(3):413-419.
60. Mee L, Honkala H, Kopra O, Vesa J, Finnila S, Visapaa I, Sang TK, Jackson GR, Salonen R, Kestila M *et al*: **Hydrolethalus syndrome is caused by a missense mutation in a novel gene HYLS1**. *Hum Mol Genet* 2005, **14**(11):1475-1488.

61. Ingraham CR, Kinoshita A, Kondo S, Yang B, Sajan S, Trout KJ, Malik MI, Dunnwald M, Goudy SL, Lovett M *et al*: **Abnormal skin, limb and craniofacial morphogenesis in mice deficient for interferon regulatory factor 6 (Irf6)**. *Nat Genet* 2006, **38**(11):1335-1340.
62. Kondo S, Schutte BC, Richardson RJ, Bjork BC, Knight AS, Watanabe Y, Howard E, de Lima RL, Daack-Hirsch S, Sander A *et al*: **Mutations in IRF6 cause Van der Woude and popliteal pterygium syndromes**. *Nat Genet* 2002, **32**(2):285-289.
63. Srichomthong C, Siriwan P, Shotelersuk V: **Significant association between IRF6 820G->A and non-syndromic cleft lip with or without cleft palate in the Thai population**. *J Med Genet* 2005, **42**(7):e46.
64. Jiang R, Lan Y, Chapman HD, Shawber C, Norton CR, Serreze DV, Weinmaster G, Gridley T: **Defects in limb, craniofacial, and thymic development in Jagged2 mutant mice**. *Genes Dev* 1998, **12**(7):1046-1057.
65. Vieira AR, Avila JR, Daack-Hirsch S, Dragan E, Felix TM, Rahimov F, Harrington J, Schultz RR, Watanabe Y, Johnson M *et al*: **Medical sequencing of candidate genes for nonsyndromic cleft lip and palate**. *PLoS Genet* 2005, **1**(6):e64.
66. Clayton-Smith J, O'Sullivan J, Daly S, Bhaskar S, Day R, Anderson B, Voss AK, Thomas T, Biesecker LG, Smith P *et al*: **Whole-exome-sequencing identifies mutations in histone acetyltransferase gene KAT6B in individuals with the Say-Barber-Biesecker variant of Ohdo syndrome**. *Am J Hum Genet* 2011, **89**(5):675-681.
67. Kraft M, Cirstea IC, Voss AK, Thomas T, Goehring I, Sheikh BN, Gordon L, Scott H, Smyth GK, Ahmadian MR *et al*: **Disruption of the histone acetyltransferase MYST4 leads to a Noonan syndrome-like phenotype and hyperactivated MAPK signaling in humans and mice**. *J Clin Invest* 2011, **121**(9):3479-3491.
68. Andelfinger G, Tapper AR, Welch RC, Vanoye CG, George AL, Jr., Benson DW: **KCNJ2 mutation results in Andersen syndrome with sex-specific cardiac and skeletal muscle phenotypes**. *Am J Hum Genet* 2002, **71**(3):663-668.
69. Yoon G, Oberoi S, Tristani-Firouzi M, Etheridge SP, Quitania L, Kramer JH, Miller BL, Fu YH, Ptacek LJ: **Andersen-Tawil syndrome: prospective cohort analysis and expansion of the phenotype**. *Am J Med Genet A* 2006, **140**(4):312-321.
70. Lindgren AM, Hoyos T, Talkowski ME, Hanscom C, Blumenthal I, Chiang C, Ernst C, Pereira S, Ordulu Z, Clericuzio C *et al*: **Haploinsufficiency of KDM6A is associated with severe psychomotor retardation, global growth restriction, seizures and cleft palate**. *Human genetics* 2013, **132**(5):537-552.
71. Putoux A, Thomas S, Coene KL, Davis EE, Alanay Y, Ogur G, Uz E, Buzas D, Gomes C, Patrier S *et al*: **KIF7 mutations cause fetal hydrolethalus and acrocallosal syndromes**. *Nature genetics* 2011, **43**(6):601-606.
72. Ng SB, Bigham AW, Buckingham KJ, Hannibal MC, McMillin MJ, Gildersleeve HI, Beck AE, Tabor HK, Cooper GM, Mefford HC *et al*: **Exome sequencing identifies MLL2 mutations as a cause of Kabuki syndrome**. *Nat Genet* 2010, **42**(9):790-793.
73. Richieri-Costa A: **Antecubital pterygium and cleft lip/palate presenting as signs of the nail-patella syndrome: report of a Brazilian family**. *American journal of medical genetics* 1991, **38**(1):9-12.
74. Beaty TH, Murray JC, Marazita ML, Munger RG, Ruczinski I, Hetmanski JB, Liang KY, Wu T, Murray T, Fallin MD *et al*: **A genome-wide association study of cleft lip with and without cleft palate identifies risk variants near MAFB and ABCA4**. *Nat Genet* 2010, **42**(6):525-529.
75. Yuan Q, Blanton SH, Hecht JT: **Association of ABCA4 and MAFB with non-syndromic cleft lip with or without cleft palate**. *Am J Med Genet A* 2011, **155A**(6):1469-1471.

76. Mohamed JY, Faqeih E, Alsiddiky A, Alshammari MJ, Ibrahim NA, Alkuraya FS: **Mutations in MEOX1, encoding mesenchyme homeobox 1, cause Klippel-Feil anomaly**. *Am J Hum Genet* 2013, **92**(1):157-161.
77. Quaderi NA, Schweiger S, Gaudenz K, Franco B, Rugarli EI, Berger W, Feldman GJ, Volta M, Andolfi G, Gilgenkrantz S *et al*: **Opitz G/BBB syndrome, a defect of midline development, is due to mutations in a new RING finger gene on Xp22**. *Nat Genet* 1997, **17**(3):285-291.
78. So J, Suckow V, Kijas Z, Kalscheuer V, Moser B, Winter J, Baars M, Firth H, Lunt P, Hamel B *et al*: **Mild phenotypes in a series of patients with Opitz GBBB syndrome with MID1 mutations**. *Am J Med Genet A* 2005, **132A**(1):1-7.
79. Jezewski PA, Vieira AR, Nishimura C, Ludwig B, Johnson M, O'Brien SE, Daack-Hirsch S, Schultz RE, Weber A, Nepomucena B *et al*: **Complete sequencing shows a role for MSX1 in non-syndromic cleft lip and palate**. *J Med Genet* 2003, **40**(6):399-407.
80. Prescott NJ, Winter RM, Malcolm S: **Maternal MTHFR genotype contributes to the risk of non-syndromic cleft lip and palate**. *J Med Genet* 2002, **39**(5):368-369.
81. Zhao M, Ren Y, Shen L, Zhang Y, Zhou B: **Association between MTHFR C677T and A1298C polymorphisms and NSCL/P risk in Asians: a meta-analysis**. *PloS one* 2014, **9**(3):e88242.
82. Martinelli M, Di Stazio M, Scapoli L, Marchesini J, Di Bari F, Pezzetti F, Carinci F, Palmieri A, Carinci P, Savoia A: **Cleft lip with or without cleft palate: implication of the heavy chain of non-muscle myosin IIA**. *J Med Genet* 2007, **44**(6):387-392.
83. Seemanova E, Passarge E, Beneskova D, Houstek J, Kasal P, Sevcikova M: **Familial microcephaly with normal intelligence, immunodeficiency, and risk for lymphoreticular malignancies: a new autosomal recessive disorder**. *American journal of medical genetics* 1985, **20**(4):639-648.
84. Krantz ID, McCallum J, DeScipio C, Kaur M, Gillis LA, Yaeger D, Jukofsky L, Wasserman N, Bottani A, Morris CA *et al*: **Cornelia de Lange syndrome is caused by mutations in NIPBL, the human homolog of Drosophila melanogaster Nipped-B**. *Nat Genet* 2004, **36**(6):631-635.
85. Tonkin ET, Wang TJ, Lisgo S, Bamshad MJ, Strachan T: **NIPBL, encoding a homolog of fungal Scc2-type sister chromatid cohesion proteins and fly Nipped-B, is mutated in Cornelia de Lange syndrome**. *Nat Genet* 2004, **36**(6):636-641.
86. Thauvin-Robinet C, Cossee M, Cormier-Daire V, Van Maldergem L, Toutain A, Alembik Y, Bieth E, Layet V, Parent P, David A *et al*: **Clinical, molecular, and genotype-phenotype correlation studies from 25 cases of oral-facial-digital syndrome type 1: a French and Belgian collaborative study**. *J Med Genet* 2006, **43**(1):54-61.
87. Zielinski D, Markus B, Sheikh M, Gymrek M, Chu C, Zaks M, Srinivasan B, Hoffman JD, Aizenbud D, Erlich Y: **OTX2 duplication is implicated in hemifacial microsomia**. *PloS one* 2014, **9**(5):e96788.
88. Hyon C, Marlin S, Chantot-Bastaraud S, Mabboux P, Beaujard MP, Al Ageeli E, Vazquez MP, Picard A, Siffroi JP, Portnoi MF: **A new 17p13.3 microduplication including the PAFAH1B1 and YWHAE genes resulting from an unbalanced X;17 translocation**. *European journal of medical genetics* 2011, **54**(3):287-291.
89. Butali A, Suzuki S, Cooper ME, Mansilla AM, Cuenco K, Leslie EJ, Suzuki Y, Niimi T, Yamamoto M, Ayanga G *et al*: **Replication of genome wide association identified candidate genes confirm the role of common and rare variants in PAX7 and VAX1 in the etiology of nonsyndromic CL(P)**. *Am J Med Genet A* 2013, **161A**(5):965-972.
90. Ichikawa E, Watanabe A, Nakano Y, Akita S, Hirano A, Kinoshita A, Kondo S, Kishino T, Uchiyama T, Niikawa N *et al*: **PAX9 and TGFB3 are linked to susceptibility to nonsyndromic cleft lip with or without cleft palate in the Japanese: population-based and family-based candidate gene analyses**. *Journal of human genetics* 2006, **51**(1):38-46.
91. Choi SJ, Marazita ML, Hart PS, Sulima PP, Field LL, McHenry TG, Govil M, Cooper ME, Letra A, Menezes R *et al*: **The PDGF-C regulatory region SNP rs28999109 decreases promoter**

transcriptional activity and is associated with CL/P. *European journal of human genetics : EJHG* 2009, **17**(6):774-784.

92. Laumonnier F, Holbert S, Ronce N, Faravelli F, Lenzner S, Schwartz CE, Lespinasse J, Van Esch H, Lacombe D, Goizet C *et al*: **Mutations in PHF8 are associated with X linked mental retardation and cleft lip/cleft palate**. *J Med Genet* 2005, **42**(10):780-786.
93. Kalscheuer VM, Freude K, Musante L, Jensen LR, Yntema HG, Gecz J, Sefiani A, Hoffmann K, Moser B, Haas S *et al*: **Mutations in the polyglutamine binding protein 1 gene cause X-linked mental retardation**. *Nat Genet* 2003, **35**(4):313-315.
94. Yang T, Jia Z, Bryant-Pike W, Chandrasekhar A, Murray JC, Fritzsch B, Bassuk AG: **Analysis of PRICKLE1 in human cleft palate and mouse development demonstrates rare and common variants involved in human malformations**. *Molecular genetics & genomic medicine* 2014, **2**(2):138-151.
95. Hahn H, Wicking C, Zaphiropoulous PG, Gailani MR, Shanley S, Chidambaram A, Vorechovsky I, Holmberg E, Unden AB, Gillies S *et al*: **Mutations of the human homolog of Drosophila patched in the nevoid basal cell carcinoma syndrome**. *Cell* 1996, **85**(6):841-851.
96. Johnson RL, Rothman AL, Xie J, Goodrich LV, Bare JW, Bonifas JM, Quinn AG, Myers RM, Cox DR, Epstein EH, Jr. *et al*: **Human homolog of patched, a candidate gene for the basal cell nevus syndrome**. *Science* 1996, **272**(5268):1668-1671.
97. Sozen MA, Suzuki K, Tolarova MM, Bustos T, Fernandez Iglesias JE, Spritz RA: **Mutation of PVRL1 is associated with sporadic, non-syndromic cleft lip/palate in northern Venezuela**. *Nat Genet* 2001, **29**(2):141-142.
98. Suzuki K, Hu D, Bustos T, Zlotogora J, Richieri-Costa A, Helms JA, Spritz RA: **Mutations of PVRL1, encoding a cell-cell adhesion molecule/herpesvirus receptor, in cleft lip/palate-ectodermal dysplasia**. *Nat Genet* 2000, **25**(4):427-430.
99. Brunetti-Pierri N, Del Gaudio D, Peters H, Justino H, Ott CE, Mundlos S, Bacino CA: **Robinow syndrome: phenotypic variability in a family with a novel intragenic ROR2 mutation**. *Am J Med Genet A* 2008, **146A**(21):2804-2809.
100. FitzPatrick DR, Carr IM, McLaren L, Leek JP, Wightman P, Williamson K, Gautier P, McGill N, Hayward C, Firth H *et al*: **Identification of SATB2 as the cleft palate gene on 2q32-q33**. *Hum Mol Genet* 2003, **12**(19):2491-2501.
101. Bernier FP, Caluseriu O, Ng S, Schwartzentruber J, Buckingham KJ, Innes AM, Jabs EW, Innis JW, Schuette JL, Gorski JL *et al*: **Haploinsufficiency of SF3B4, a component of the pre-mRNA spliceosomal complex, causes Nager syndrome**. *Am J Hum Genet* 2012, **90**(5):925-933.
102. Roessler E, Belloni E, Gaudenz K, Jay P, Berta P, Scherer SW, Tsui LC, Muenke M: **Mutations in the human Sonic Hedgehog gene cause holoprosencephaly**. *Nat Genet* 1996, **14**(3):357-360.
103. Prescott NJ, Lees MM, Winter RM, Malcolm S: **Identification of susceptibility loci for nonsyndromic cleft lip with or without cleft palate in a two stage genome scan of affected sib-pairs**. *Human genetics* 2000, **106**(3):345-350.
104. Hastbacka J, de la Chapelle A, Mahtani MM, Clines G, Reeve-Daly MP, Daly M, Hamilton BA, Kusumi K, Trivedi B, Weaver A *et al*: **The diastrophic dysplasia gene encodes a novel sulfate transporter: positional cloning by fine-structure linkage disequilibrium mapping**. *Cell* 1994, **78**(6):1073-1087.
105. Benko S, Fantes JA, Amiel J, Kleinjan DJ, Thomas S, Ramsay J, Jamshidi N, Essafi A, Heaney S, Gordon CT *et al*: **Highly conserved non-coding elements on either side of SOX9 associated with Pierre Robin sequence**. *Nat Genet* 2009, **41**(3):359-364.
106. Foster JW, Dominguez-Steglich MA, Guioli S, Kwok C, Weller PA, Stevanovic M, Weissenbach J, Mansour S, Young ID, Goodfellow PN *et al*: **Campomelic dysplasia and autosomal sex reversal caused by mutations in an SRY-related gene**. *Nature* 1994, **372**(6506):525-530.

107. Pop R, Zaragoza MV, Gaudette M, Dohrmann U, Scherer G: **A homozygous nonsense mutation in SOX9 in the dominant disorder campomelic dysplasia: a case of mitotic gene conversion**. *Human genetics* 2005, **117**(1):43-53.
108. Wagner T, Wirth J, Meyer J, Zabel B, Held M, Zimmer J, Pasantes J, Bricarelli FD, Keutel J, Hustert E *et al*: **Autosomal sex reversal and campomelic dysplasia are caused by mutations in and around the SRY-related gene SOX9**. *Cell* 1994, **79**(6):1111-1120.
109. Czako M, Riegel M, Morava E, Bajnoczky K, Kosztolanyi G: **Opitz "C" trigonocephaly-like syndrome in a patient with terminal deletion of 2p and partial duplication of 17q**. *Am J Med Genet A* 2004, **131**(3):310-312.
110. Alkuraya FS, Saadi I, Lund JJ, Turbe-Doan A, Morton CC, Maas RL: **SUMO1 haploinsufficiency leads to cleft lip and palate**. *Science* 2006, **313**(5794):1751.
111. Song T, Li G, Jing G, Jiao X, Shi J, Zhang B, Wang L, Ye X, Cao F: **SUMO1 polymorphisms are associated with non-syndromic cleft lip with or without cleft palate**. *Biochemical and biophysical research communications* 2008, **377**(4):1265-1268.
112. Packham EA, Brook JD: **T-box genes in human disorders**. *Hum Mol Genet* 2003, **12 Spec No 1**:R37-44.
113. Herman SB, Guo T, McGinn DM, Blonska A, Shanske AL, Bassett AS, Chow EW, Bowser M, Sheridan M, Beemer F *et al*: **Overt cleft palate phenotype and TBX1 genotype correlations in velo-cardio-facial/DiGeorge/22q11.2 deletion syndrome patients**. *Am J Med Genet A* 2012, **158A**(11):2781-2787.
114. Kobrynski LJ, Sullivan KE: **Velocardiofacial syndrome, DiGeorge syndrome: the chromosome 22q11.2 deletion syndromes**. *Lancet* 2007, **370**(9596):1443-1452.
115. Andreou AM, Pauws E, Jones MC, Singh MK, Bussen M, Doudney K, Moore GE, Kispert A, Brosens JJ, Stanier P: **TBX22 missense mutations found in patients with X-linked cleft palate affect DNA binding, sumoylation, and transcriptional repression**. *Am J Hum Genet* 2007, **81**(4):700-712.
116. Braybrook C, Doudney K, Marcano AC, Arnason A, Bjornsson A, Patton MA, Goodfellow PJ, Moore GE, Stanier P: **The T-box transcription factor gene TBX22 is mutated in X-linked cleft palate and ankyloglossia**. *Nat Genet* 2001, **29**(2):179-183.
117. Braybrook C, Lisgo S, Doudney K, Henderson D, Marcano AC, Strachan T, Patton MA, Villard L, Moore GE, Stanier P *et al*: **Craniofacial expression of human and murine TBX22 correlates with the cleft palate and ankyloglossia phenotype observed in CPX patients**. *Hum Mol Genet* 2002, **11**(22):2793-2804.
118. Marcano AC, Doudney K, Braybrook C, Squires R, Patton MA, Lees MM, Richieri-Costa A, Lidral AC, Murray JC, Moore GE *et al*: **TBX22 mutations are a frequent cause of cleft palate**. *J Med Genet* 2004, **41**(1):68-74.
119. **Positional cloning of a gene involved in the pathogenesis of Treacher Collins syndrome. The Treacher Collins Syndrome Collaborative Group**. *Nat Genet* 1996, **12**(2):130-136.
120. Milunsky JM, Maher TA, Zhao G, Roberts AE, Stalker HJ, Zori RT, Burch MN, Clemens M, Mulliken JB, Smith R *et al*: **TFAP2A mutations result in branchio-oculo-facial syndrome**. *Am J Hum Genet* 2008, **82**(5):1171-1177.
121. Ardinger HH, Buetow KH, Bell GI, Bardach J, VanDemark DR, Murray JC: **Association of genetic variation of the transforming growth factor-alpha gene with cleft lip and palate**. *Am J Hum Genet* 1989, **45**(3):348-353.
122. Machida J, Yoshiura K, Funkhauser CD, Natsume N, Kawai T, Murray JC: **Transforming growth factor-alpha (TGFA): genomic structure, boundary sequences, and mutation analysis in nonsyndromic cleft lip/palate and cleft palate only**. *Genomics* 1999, **61**(3):237-242.

123. Shiang R, Lidral AC, Ardinger HH, Buetow KH, Romitti PA, Munger RG, Murray JC: **Association of transforming growth-factor alpha gene polymorphisms with nonsyndromic cleft palate only (CPO)**. *Am J Hum Genet* 1993, **53**(4):836-843.
124. Lidral AC, Romitti PA, Basart AM, Doetschman T, Leysens NJ, Daack-Hirsch S, Semina EV, Johnson LR, Machida J, Burds A *et al*: **Association of MSX1 and TGFB3 with nonsyndromic clefting in humans**. *Am J Hum Genet* 1998, **63**(2):557-568.
125. Loeys BL, Chen J, Neptune ER, Judge DP, Podowski M, Holm T, Meyers J, Leitch CC, Katsanis N, Sharifi N *et al*: **A syndrome of altered cardiovascular, craniofacial, neurocognitive and skeletal development caused by mutations in TGFBR1 or TGFBR2**. *Nat Genet* 2005, **37**(3):275-281.
126. Gripp KW, Wotton D, Edwards MC, Roessler E, Ades L, Meinecke P, Richieri-Costa A, Zackai EH, Massague J, Muenke M *et al*: **Mutations in TGIF cause holoprosencephaly and link NODAL signalling to human neural axis determination**. *Nat Genet* 2000, **25**(2):205-208.
127. Barbaro V, Nardiello P, Castaldo G, Willoughby CE, Ferrari S, Ponzin D, Amato F, Bonifazi E, Parekh M, Calistri A *et al*: **A novel de novo missense mutation in TP63 underlying germline mosaicism in AEC syndrome: implications for recurrence risk and prenatal diagnosis**. *Am J Med Genet A* 2012, **158A**(8):1957-1961.
128. Barrow LL, van Bokhoven H, Daack-Hirsch S, Andersen T, van Beersum SE, Gorlin R, Murray JC: **Analysis of the p63 gene in classical EEC syndrome, related syndromes, and non-syndromic orofacial clefts**. *J Med Genet* 2002, **39**(8):559-566.
129. Celli J, Duijf P, Hamel BC, Bamshad M, Kramer B, Smits AP, Newbury-Ecob R, Hennekam RC, Van Buggenhout G, van Haeringen A *et al*: **Heterozygous germline mutations in the p53 homolog p63 are the cause of EEC syndrome**. *Cell* 1999, **99**(2):143-153.
130. Giampietro PF, Baker MW, Basehore MJ, Jones JR, Seroogy CM: **Novel mutation in TP63 associated with ectrodactyly ectodermal dysplasia and clefting syndrome and T cell lymphopenia**. *Am J Med Genet A* 2013, **161A**(6):1432-1435.
131. Leoyklang P, Siriwan P, Shotelersuk V: **A mutation of the p63 gene in non-syndromic cleft lip**. *J Med Genet* 2006, **43**(6):e28.
132. McGrath JA, Duijf PH, Doetsch V, Irvine AD, de Waal R, Vanmolkot KR, Wessagowit V, Kelly A, Atherton DJ, Griffiths WA *et al*: **Hay-Wells syndrome is caused by heterozygous missense mutations in the SAM domain of p63**. *Hum Mol Genet* 2001, **10**(3):221-229.
133. van Bokhoven H, Hamel BC, Bamshad M, Sangiorgi E, Gurrieri F, Duijf PH, Vanmolkot KR, van Beusekom E, van Beersum SE, Celli J *et al*: **p63 Gene mutations in eec syndrome, limb-mammary syndrome, and isolated split hand-split foot malformation suggest a genotype-phenotype correlation**. *Am J Hum Genet* 2001, **69**(3):481-492.
134. el Ghouzzi V, Le Merrer M, Perrin-Schmitt F, Lajeunie E, Benit P, Renier D, Bourgeois P, Bolcato-Bellemin AL, Munnich A, Bonaventure J: **Mutations of the TWIST gene in the Saethre-Chotzen syndrome**. *Nat Genet* 1997, **15**(1):42-46.
135. Howard TD, Paznekas WA, Green ED, Chiang LC, Ma N, Ortiz de Luna RI, Garcia Delgado C, Gonzalez-Ramos M, Kline AD, Jabs EW: **Mutations in TWIST, a basic helix-loop-helix transcription factor, in Saethre-Chotzen syndrome**. *Nat Genet* 1997, **15**(1):36-41.
136. Andrieux J, Villenet C, Quief S, Lignon S, Geffroy S, Roumier C, de Leersnyder H, de Blois MC, Manouvrier S, Delobel B *et al*: **Genotype phenotype correlation of 30 patients with Smith-Magenis syndrome (SMS) using comparative genome hybridisation array: cleft palate in SMS is associated with larger deletions**. *J Med Genet* 2007, **44**(8):537-540.
137. Slavotinek AM, Chao R, Vacik T, Yahyavi M, Abouzeid H, Bardakjian T, Schneider A, Shaw G, Sherr EH, Lemke G *et al*: **VAX1 mutation associated with microphthalmia, corpus callosum agenesis, and orofacial clefting: the first description of a VAX1 phenotype in humans**. *Human mutation* 2012, **33**(2):364-368.

138. Ashe A, Butterfield NC, Town L, Courtney AD, Cooper AN, Ferguson C, Barry R, Olsson F, Liem KF, Jr., Parton RG *et al*: **Mutations in mouse Ift144 model the craniofacial, limb and rib defects in skeletal ciliopathies**. *Hum Mol Genet* 2012, **21**(8):1808-1823.
139. Bredrup C, Saunier S, Oud MM, Fiskerstrand T, Hoischen A, Brackman D, Leh SM, Midtbo M, Filhol E, Bole-Feysot C *et al*: **Ciliopathies with skeletal anomalies and renal insufficiency due to mutations in the IFT-A gene WDR19**. *Am J Hum Genet* 2011, **89**(5):634-643.
140. Bergemann AD, Cole F, Hirschhorn K: **The etiology of Wolf-Hirschhorn syndrome**. *Trends Genet* 2005, **21**(3):188-195.
141. Niemann S, Zhao C, Pascu F, Stahl U, Aulepp U, Niswander L, Weber JL, Muller U: **Homozygous WNT3 mutation causes tetra-amelia in a large consanguineous family**. *American journal of human genetics* 2004, **74**(3):558-563.
142. Chiquet BT, Blanton SH, Burt A, Ma D, Stal S, Mulliken JB, Hecht JT: **Variation in WNT genes is associated with non-syndromic cleft lip with or without cleft palate**. *Hum Mol Genet* 2008, **17**(14):2212-2218.
143. Herman SB, Holman SK, Robertson SP, Davidson L, Taragin B, Samanich J: **Severe osteopathia striata with cranial sclerosis in a female case with whole WTX gene deletion**. *Am J Med Genet A* 2013, **161A**(3):594-599.
144. Jenkins ZA, van Kogelenberg M, Morgan T, Jeffs A, Fukuzawa R, Pearl E, Thaller C, Hing AV, Porteous ME, Garcia-Minaur S *et al*: **Germline mutations in WTX cause a sclerosing skeletal dysplasia but do not predispose to tumorigenesis**. *Nat Genet* 2009, **41**(1):95-100.
145. Tucker ME, Escobar LF: **Cleft lip/palate associated with 17p13.3 duplication involving a single candidate gene (YWHAE)**. *Clin Genet* 2014, **85**(6):600-601.
146. Cerruti Mainardi P, Pastore G, Zweier C, Rauch A: **Mowat-Wilson syndrome and mutation in the zinc finger homeo box 1B gene: a well defined clinical entity**. *J Med Genet* 2004, **41**(2):e16.
