# Supplementary material for: MicroRNA-374a, -4680, and -133b suppress cell proliferation through the regulation of genes associated with human cleft palate in cultured human palate cells
Source: BMC Med Genomics. 2019 Jul 1;12:93. doi: 10.1186/s12920-019-0546-z (PMC6604454; doi:10.1186/s12920-019-0546-z)
Supplement: Supplementary file 8 — Table S8. Transfection efficiency of miRNA mimic and inhibitor. (PDF 51 kb) [file 12920_2019_546_MOESM8_ESM.pdf]

Table S8. Transfection efficiency of miRNA mimic and inhibitor

**mimic**		
	negative control	target miRNA
miR-133b	1 ± 0.11	66258 ± 1012***
miR-374a-5p	1 ± 0.086	296.5 ± 24.7***
miR-4680-3p	1 ± 0.073	39752 ± 5075***

**inhibitor**		
	negative control	target miRNA
miR-133b	1 ± 0.19	0.61 ± 0.014*
miR-374a-5p	1 ± 0.21	0.026 ± 0.0023***
miR-4680-3p	1 ± 0.073	0.50 ± 0.056***
